# Mitochondria in oxidative stress, inflammation and aging: from mechanisms to therapeutic advances

**DOI:** 10.1038/s41392-025-02253-4

**Published:** 2025-06-11

**Authors:** Xieyang Xu, Yan Pang, Xianqun Fan

**Affiliations:** 1 Department of Ophthalmology, Shanghai Ninth People’s Hospital, School of Medicine, Shanghai Jiao Tong University, Shanghai, China; 2 Shanghai Key Laboratory of Orbital Diseases and Ocular Oncology, Center for Basic Medical Research and Innovation in Visual System Diseases, Ministry of Education, Shanghai, China; 3 State Key Laboratory of Eye Health, Department of Ophthalmology, Shanghai Ninth People’s Hospital, Shanghai Jiao Tong University School of Medicine, Shanghai, China

**Keywords:** Cell biology, Diseases

## Abstract

Mitochondria are the energy production centers in cells and have unique genetic information. Due to the irreplaceable function of mitochondria, mitochondrial dysfunction often leads to pathological changes. Mitochondrial dysfunction induces an imbalance between oxidation and antioxidation, mitochondrial DNA (mtDNA) damage, mitochondrial dynamics dysregulation, and changes in mitophagy. It results in oxidative stress due to excessive reactive oxygen species (ROS) generation, which contributes to cell damage and death. Mitochondrial dysfunction can also trigger inflammation through the activation of damage-associated molecular patterns (DAMPs), inflammasomes and inflammatory cells. Besides, mitochondrial alterations in the functional regulation, energy metabolism and genetic stability accompany the aging process, and there has been a lot of evidence suggesting that oxidative stress and inflammation, both of which are associated with mitochondrial dysfunction, are predisposing factors of aging. Therefore, this review hypothesizes that mitochondria serve as central hubs regulating oxidative stress, inflammation, and aging, and their dysfunction contributes to various diseases, including cancers, cardiovascular diseases, neurodegenerative disorders, metabolic diseases, sepsis, ocular pathologies, liver diseases, and autoimmune conditions. Moreover, we outline therapies aimed at various mitochondrial dysfunctions, highlighting their performance in animal models and human trials. Additionally, we focus on the limitations of mitochondrial therapy in clinical applications, and discuss potential future research directions for mitochondrial therapy.

## Introduction

Ever since mitochondria were first discovered in muscle cells in the 19th century, there has been an increasing amount of research in the biological and medical fields that focus on the homeostasis of mitochondria and its dysregulation in diseases. Mitochondria are bilayer-coated organelles in cells and are usually known to be the main energy-producing sites within cells.^1,2^ In general, the number of mitochondria is in direct portion to the metabolic rates. With glucose metabolizing into pyruvate, it enters the mitochondria and participates in the tricarboxylic acid cycle (TCA), where reduced nicotinamide adenine dinucleotide (NADH) and reduced flavin adenine dinucleotide (FADH_2_) are produced and then go through the respiratory chain pushing the adenosine triphosphate (ATP) pump to produce large amounts of ATP. During this OXPHOS process, some electrons leak to oxygen, generating superoxide anion (O₂⁻), hydrogen peroxide (H₂O₂), and other forms of oxidative products. These oxidative products could be referred to as ROS. Generally, there is a corresponding antioxidant system to equilibrate oxidation. Antioxidant enzymes, such as catalase and superoxide dismutase (SOD), attribute to the decomposition and conversion of ROS which help decrease ROS accumulation. However, extreme oxidation breaks the balance between the oxidative and antioxidant systems, resulting in oxidative stress and an excessive accumulation of ROS.^3,4^ ROS accumulation induces damage to cellular components such as DNA, RNA, and proteins.^5–7^ It also influences pyroptosis through the mediation of NOD-like receptor and pyrin domain containing-3 (NLRP3) inflammasome in response to certain stimuli.^8^ ROS triggered by overload of intracellular iron metabolism regulate cell death and cause senescence, which is the key mechanism of ferroptosis.^9,10^ In general, oxidative stress leads to cell damage and induces pathological conditions.

Mitochondria are important mediators in the occurrence and development of inflammation.^11,12^ Inflammation is a series of defensive responses to stimuli that may be related to viral or bacterial infections, autoimmune diseases and tumors.^13,14^ However, when inflammation overreacts, it will inevitably trigger autoimmune diseases. In this regard, oxidative stress can activate DAMPs, primarily in the form of mtDNA, or indirectly trigger inflammasome activation. Furthermore, oxidative stress influences the regulation, differentiation, and function of inflammatory cells, thereby modulating the occurrence of immune inflammatory response.^15^ Mitochondria are involved in the regulation of inflammation and immunity through oxidative stress and ROS levels.^11,16,17^

Aging is a sophisticated dysregulation of multiple organs raised by both intrinsic and extrinsic factors. Increasingly hallmarks have been found to be associated with aging. In 2023, Lopez-Otin proposed twelve markers of aging, including genomic instability, telomere loss, epigenetic changes, loss of protein balance, failure of macroautophagy, dysregulated nutrient sensing, mitochondrial dysfunction, cell senescence, stem cell depletion, altered intercellular communication, chronic inflammation, and dysbiosis.^18^ Among these factors, mitochondria dysfunction is regarded as one of the tremendous determinants in driving aging.^19,20^ With aging, mitochondrial function declines, leading to decreased energy metabolism, increased ROS production, and the accumulation of mtDNA mutations, alongside the deterioration of other mitochondrial functions. Furthermore, oxidative stress and inflammatory pathways triggered by mitochondrial dysfunction also have a crucial impact on aging. Consequently, mitochondria may play a central regulatory role between oxidative stress, inflammation, and aging.

In these ways, mitochondria contribute to various diseases, including tumors, cardiovascular diseases, neurodegenerative diseases, metabolic abnormalities, ocular diseases, liver diseases, and autoimmune diseases, by inducing mitochondrial dysfunction or through regulating oxidative stress, inflammation, and aging.^21–24^ As mitochondria are responsible for energy production and cellular respiration, their dysfunction will result in muscle diseases, such as mitochondrial myopathies and myotonic dystrophy.^25,26^ Cardiovascular diseases like cardiomyopathy, and neurodegenerative diseases such as Alzheimer’s disease, can be attributed to abnormal energy supply. Mitochondrial dysfunction in calcium homeostasis is associated with an increased risk of heart failure and atrial fibrillation.^27,28^ Mitochondria participate in glycolysis, fatty acid oxidation, and amino acid metabolism. Disruptions in these metabolic pathways result in metabolic disorders, contributing to diabetes, obesity, and fatty liver disease.^29,30^ Additionally, mutations in mtDNA lead to mitochondrial inherited diseases. Furthermore, by altering energy metabolism, modulating tumor immunity, and shaping the tumor microenvironment, mitochondria influence tumor development and progression. In general, given the complex functions of mitochondria, any dysfunction may result in abnormal pathological states, contributing to the development of numerous diseases.

**Fig. 1:**
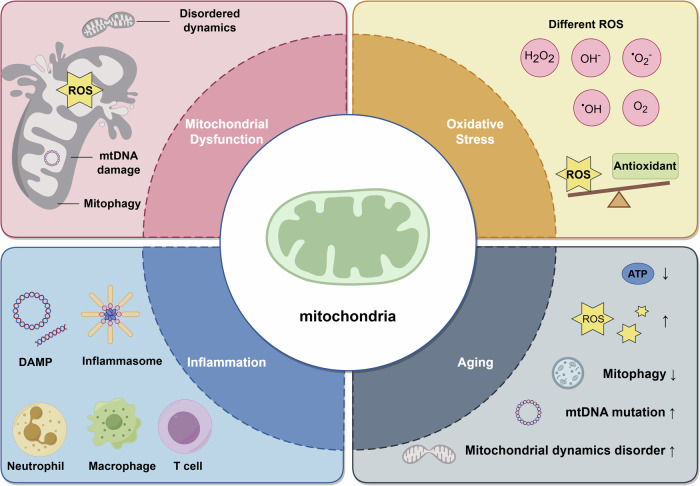
Mitochondria act as the core to link inflammation, oxidative stress, and aging. When mitochondrial dysfunction occurs via disturbing oxidation and antioxidation, mitochondrial dynamics, mitochondrial genome and mitophagy, it’s capable of triggering a cascade of chain reactions. The imbalance between oxidation and antioxidation contributes to oxidative stress, which, in turn, exacerbates mitochondrial dysfunction in a vicious cycle. Under certain stress conditions, mitochondria release mtDNA, mtRNA, ROS, and other molecules to activate inflammasomes, and to act as DAMPs, triggering downstream inflammatory signal pathways. In addition, mitochondrial dysfunction modulates the functions of neutrophils, macrophages, and T cells, thus facilitating inflammation in a series of approaches. Previous evidence has shown that mitochondrial dysfunction is one of the hallmarks of aging, manifested as impaired mitophagy, mtDNA damage, disrupted mitochondrial dynamics, and metabolic imbalances in senescent cells. Additionally, oxidative stress and inflammation have been proven to play a role in the aging process, making mitochondrial dysfunction a direct as well as indirect contributor to aging. In summary, due to its central role in metabolism and signaling transduction, mitochondria serve as a pivotal hub, linking oxidative stress, inflammation, and aging, and mediating their complex interactions. ROS reactive oxygen species, mtDNA mitochondrial DNA, DAMPs damage-associated molecular patterns. This figure was created using Figdraw

Although some have focused on the interrelationships between mitochondria and oxidative stress, mitochondria and inflammation, and mitochondria and aging, there are few researches that summarize how mitochondria tie these three together. Considering the critical role of mitochondria, we outline mitochondrial dysfunction in oxidation and antioxidation, mitochondrial dynamics, mtDNA damage, and mitophagy. We also summarize innovative researches on the role of mitochondria in oxidative stress, inflammation, and aging, while exploring the interconnections among these factors. In addition, diseases related to mitochondrial dysfunction as well as therapies targeting mitochondria are also included. An in-depth comprehension of the interplay between mitochondrial dysfunction and oxidative stress, inflammation, and aging, along with their impacts in disease and therapies, will enhance understanding of these diseases and make better clinical decisions (Fig. 1).

## Mitochondrial dysfunction

### Imbalance of oxidation and antioxidation

**Fig. 2:**
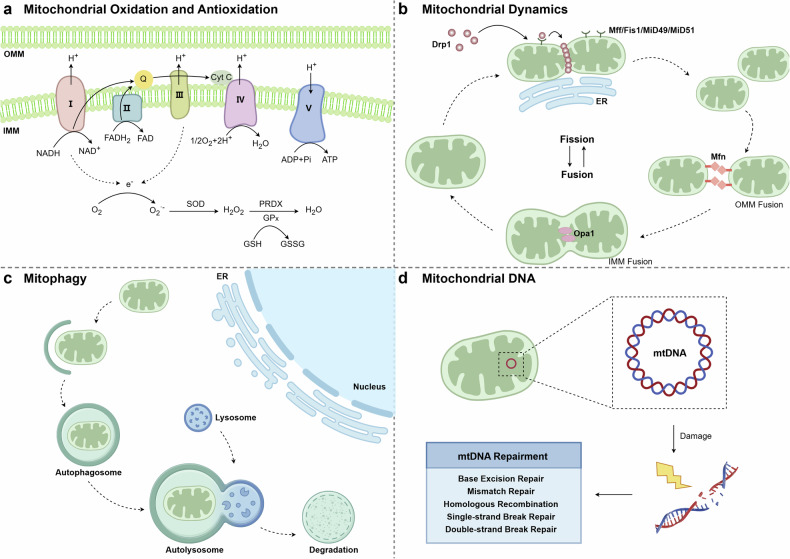
The diagram illustrating the fundamental functions of mitochondria. **a** In mitochondrial oxidation and antioxidation, ROS are primarily produced as byproducts of the respiratory chain. These ROS are cleared by antioxidant enzymes such as SOD, GPx, and PRDX. **b** Mitochondrial dynamics involve mitochondrial fission and fusion. Mitochondrial fission is primarily initiated by Drp1, which binds to Mff, Fis1, Mid49, and Mid51 on the outer mitochondrial membrane, forming a ring around the mitochondria to facilitate their division. Mitochondrial fusion is mediated by Mfn, responsible for outer membrane fusion, and Opa1, which facilitates inner membrane fusion respectively. **c** When mitochondria are damaged and recognized, mitophagy is initiated. Damaged mitochondria are encapsulated by autophagosomes and subsequently fuse with lysosomes to form autolysosomes. Mitochondria are completely degraded in autolysosomes. **d** In mitochondrial DNA, the composition of mtDNA and the primary mechanisms of mtDNA damage repairment are demonstrated. ROS, reactive oxygen species. mtDNA, mitochondrial DNA. SOD super oxide dismutase, GPx glutathione peroxidase, PRDX peroxiredoxin, Mff mitochondrial fission factor, Fis1 fission protein 1, Mid49/51 mitochondrial dynamics protein 49/51, Mfn mitofusin, Opa1 optic atrophy 1, ER endoplasmic reticulum. This figure was created using Figdraw

Mitochondrial ROS produced through the respiratory chain is the major source of ROS (Fig. 2a). The respiratory chain consists of a cluster of complexes located in the mitochondrial membrane that receive hydrogen ions from NADH or FADH_2_ and transfer electrons to oxygen, resulting in H_2_O formation as well as ROS generation.^31^ Complex I and complex III are the main sites of ROS generation in mitochondria.^32^ In complex I, the oxidation of NADH produces electrons, then these electrons are delivered to oxygen and produce O_2_^•−^.^33^ Subsequently, in complex III, O_2_ interacts with semiquinone, binding to the Qo site and producing large amounts of O_2_^•−^.^34^ O_2_^•−^ produced in complexes I and complex III will be converted to H_2_O_2_ by SOD, then it will be transmitted to the cytoplasm and participate in other biological reactions. In addition, O_2_^•−^ or H_2_O_2_ can also be produced at other sites in the mitochondria, such as some mitochondrial enzymes, including glycerol 3-phosphate dehydrogenase and electron transferring flavoprotein-Q oxidoreductase.^35^ Therefore, it is worth exploring other sites of ROS production in mitochondria.

To deal with the extra ROS, mitochondria developed an antioxidant system to counteract the massive buildup of ROS and avoid oxidative stress. Typically, one of the mitochondrial antioxidant mechanisms is the glutathione (GSH) system.^36^ Mechanistically, GSH can be converted into oxidized glutathione (GSSG) under the catalysis of glutathione peroxidase (GPx), at the same time H_2_O_2_ is able to transfer into non-toxic H_2_O, then GSSG will be reduced to reform GSH again by glutathione reductase to maintain a cycle. GSH is usually manufactured in the cytoplasm and then transported into organelles. After that, it passes through the mitochondrial outer membrane readily and then through the inner membrane mediated by GSH transporters.^37^ The member 39 in solute carrier family 25 (SLC25A39) acts as the mitochondrial transporter required for the import of mitochondrial GSH which can maintain the mitochondrial GSH concentration in mammalian cells.^38^ Accordingly, the mitochondrial GSH was reduced in *SLC25A39*-knockout cells, while the global GSH level was not affected.^38^

In addition to the GSH system, several other enzymes aid in peroxide metabolism. SOD exerts antioxidant roles by catalyzing the transformation of O_2_^•−^ into H_2_O_2_, which can be scavenged to H_2_O via enzymes such as peroxiredoxin. Catalase scavenges hydrogen peroxide by stimulating the decomposition of H_2_O_2_ to H_2_O and O_2_.^39^ Except for enzymes, some substances are also potent mitochondrial antioxidants. For example, melatonin can not only directly clean ROS but also promote antioxidant enzymes such as GPx and glutathione reductase, thereby indirectly scavenging ROS.^40^

### Mitochondrial dynamics

Mitochondria, as a kind of double-membrane-coated organelle, can dynamically be fused or split under specific conditions. Mitochondrial dynamics is identified as the fusion and fission of mitochondria (Fig. 2b).^41–43^ It endows mitochondria with different shapes accompanied by changes in quantity and quality of mitochondria, thus finally affecting cell metabolism and function.

Alterations in mitochondrial dynamics depend on several mitochondrial proteins.^44^ For example, dynamin-related protein 1 (Drp1) is a major protein involved in mitochondrial fission. In mitochondrial fission, Drp1 is anchored to the outer mitochondrial membrane by the recruiting of mitochondrial fission factor (Mff), fission protein 1 (Fis1), mitochondrial dynamics protein 49 (MiD49), and mitochondrial dynamics protein 51 (MiD51).^45,46^ These proteins are all located in the outer mitochondrial membrane. Mff is the primary recruiting protein of Drp1. It can interact with Drp1 and promote Drp1 accumulation at the mitochondrial membrane.^47^ Fis1, MiD49 and MiD51 are also involved in Drp1 recruitment.^46,48,49^ In recent years, it has been found that mitochondrial fission can be divided into midzone fission and periphery fission, Mff is enriched in midzone and Fis1 in periphery zone.^50^ Drp1 is a GTPase, facilitating membrane curving and contraction by hydrolyzing GTP.^51^ After being recruited, Drp1 aggregates at fission sites and forms a ring or spiral structure around the outer mitochondrial membrane.^52^ As the Drp1 ring continues to contract, the outer mitochondrial membrane eventually splits to form two separate small mitochondria.

Mitochondrial fusion is related to three proteins, optic atrophy 1 (OPA1), mitofusin 1 (Mfn1), and mitofusin 2 (Mfn2). Mfn1 and Mfn2 regulate the fusion of the outer mitochondrial membrane which occurs earlier, whereas OPA1 is normally associated with the fusion of the inner mitochondrial membrane.^53^ When two mitochondria get closer, Mfn1 or Mfn2 interact between the outer membranes of the two mitochondria. Two Mfn proteins recognize each other and form a dimer that links the two mitochondrial outer membranes.^54,55^ Opa1 is localized in the inner mitochondrial membrane, which is responsible for the fusion of the inner membrane, and maintains the membrane structure.^56–58^

The homeostasis of mitochondrial fission and fusion is involved in the quality control of mitochondria and the regulation of mitochondrial function. An imbalance in either would lead to mitochondrial dysfunction, which affects the maintenance of cell homeostasis and causes illness.

### mtDNA damage

The most striking difference between mitochondria and other organelles is that they have a separate set of genetic materials (mtDNA) consisting of 13 protein-coding genes, 22 tRNA genes, and 2 rRNA genes, which assist in maintaining mitochondrial function.^59^ mtDNA-encoded proteins are mainly involved in the respiratory chain and are therefore key components of cellular energy production. mtDNA is essential for maintaining mitochondrial function, but it is more susceptible to being damaged by ROS than nuclear DNA since it is closer to the mitochondrial ROS production site, after which mitochondrial genome stability is influenced and diseases occur. There are a variety of countermeasures to mtDNA damage (Fig. 2d).^60^ mtDNA repair is partially similar to the repair pathway of nuclear DNA. mtDNA repair methods include base excision repair, mismatch repair, single-strand break repair, double-strand break repair, and homologous recombination.^61^^,^^62^ Mitochondria can also directly remove the damaged mtDNA fragments to the cytoplasm in certain conditions. Mitochondrial quality control, such as mitophagy and mitochondrial dynamics, are also involved in mtDNA regulation. Mitophagy can directly clear the damaged mitochondria if it is not able to be repaired. Mitochondrial dynamics control the copy number of mtDNA, while the maintenance of mtDNA copy number is attributed to mitochondrial fusion.^63,64^ It has been found that Opa1-Exon4b specifically binds to the D-loop of mtDNA accompanied with increased mtDNA transcription and subsequently repair the mitochondrial respiration.^65^

Despite efforts have been made to repair mtDNA with various mechanisms, the mutation rate of mtDNA remains higher than nuclear DNA. The resulted mtDNA damage will lead to mitochondrial dysfunction by affecting respiration activity and metabolism.

### Mitophagy

**Fig. 3:**
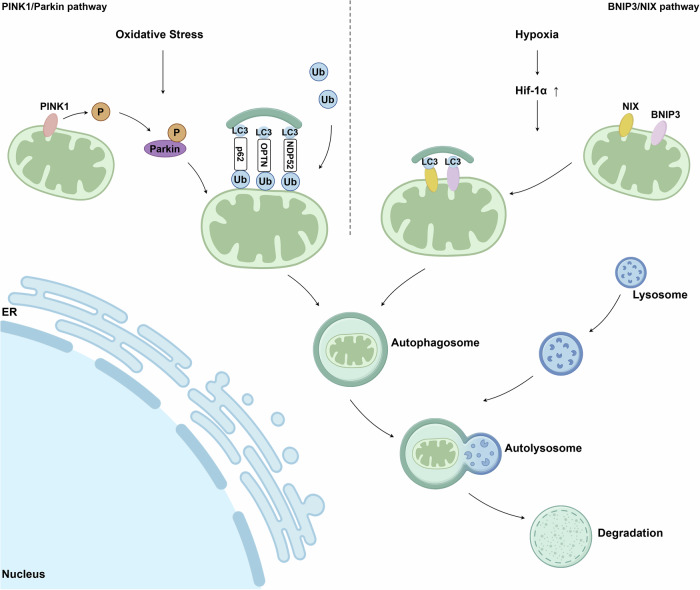
Two main mitophagy pathways, including the PINK1/Parkin pathway and the BNIP3/NIX pathway. PINK1 PTEN-induced kinase 1, Ub ubiquitin, P phosphorylation, LC3 microtubule-associated protein light chain 3, BNIP3 Bcl2/adenovirus E1B 19 kDa interacting protein 3, NIX BNIP3-like, ER endoplasmic reticulum. This figure was created using Figdraw

Mitophagy is a mechanism to clear damaged mitochondria (Fig. 2c, Fig. 3). When mitochondria are impaired in some physiological or pathological conditions with mtDNA damage and mitochondrial dysfunction, mitophagy can remove damaged mitochondria to maintain cell homeostasis.

Specifically, the PTEN-induced kinase 1 (PINK1)/Parkin pathway is a classic mechanism that activates mitophagy by mediating the recognition of damaged mitochondria.^66^ Damaged mitochondria induce PINK1 to bind with the translocase of the outer membrane, prompting phosphorylation of Parkin and ubiquitin, labeling damaged mitochondria, and mediating mitophagy.^67^

Alternatively, the damaged mitochondria can also be removed via the Bcl2/adenovirus E1B 19 kDa interacting protein 3 (BNIP3)/NIX (also known as BNIP3-like) pathway. Mitophagy induced by the BNIP3/NIX pathway may be related to the initiation of mitochondrial depolarization or the recruitment of autophagy components.^68^ Of note, the BNIP3/NIX pathway was specifically up-regulated when mitochondrial oxidative stress was generated in the hypoxic environment to further induce mitophagy.^69^

Hypoxia-inducible factor alpha (HIF-1α) is an upstream signal of BNIP3 and mediates mitophagy through the key HIF-1α/BNIP3 pathway in the process of mitophagy. HIF-1α is usually activated under hypoxic conditions and then regulates the expression of multiple genes, thereby altering the physiological processes of the body under hypoxic conditions. ROS in hypoxia can increase the stability of HIF-1α and induce the activation of HIF -1α.^70,71^ The regulation of ROS by HIF-1α is usually negative. HIF-1α suppresses ROS generation by enhancing glycolysis, inhibiting the TCA, and promoting an upregulation in antioxidants.^72–74^ Although it is well known that HIF-1α decreases ROS production, Chen et al. proposed the HIF-1α/ROS/VEGF pathway.^75^ In human umbilical vein endothelial cells, treatment with the HIF-1α inhibitor and melatonin lowered ROS release and vascular endothelial growth factor (VEGF) expression.^75^ HIF-1α regulates mitophagy through the downstream factor BNIP3. In the hypoxic environment, activation of the HIF-1α/BNIP3 pathway induced BNIP3-associated mitophagy.^76^ In contrast, silencing and down-regulation of cellular BNIP3 levels by siRNA inhibited mitophagy, which led to an increasing ROS level and a decreased protein level of the antioxidant MnSOD.^76^ In a cell experiment, when renal tubular cells experienced hypoxia and then reoxygenation, the levels of HIF-1α and BNIP3 were increased, mediating mitophagy, which could protect cells from cell apoptosis.^77^

Once the damaged mitochondria are sensed, the autophagosomes engulf mitochondria and guide them towards the lysosome for degradation.^78^ Autophagosomes in mitochondria depends on the microtubule-associated protein light chain 3 (LC3)/GABA(A) receptor-associated protein (GABARAP) family.^79,80^ ATPase family AAA domain-containing protein 3B (ATAD3B) is a mitophagy receptor. When it comes to oxidative stress, ATAD3B is situated in the mitochondrial membrane and functions as recruiting LC3, which will promote mitophagy.^81^

## Mitochondria and oxidative stress

Mitochondria are closely associated with oxidative stress as mitochondria act as the sites of ROS generation and clearance. Accordingly, oxidative stress is the key threat to mitochondrial damage. Extensive evidences have highlighted the interaction between mitochondrial metabolic dysfunction, mtDNA damage, mitochondrial dynamics disorders, mitophagy dysregulation, and oxidative stress, while targeting oxidative stress represents a promising strategy for combating mitochondria diseases.

### Excessive ROS triggers oxidative stress

Although ROS production in mitochondria is in balance with an intracellular antioxidant system under physiological conditions, the balance is easy to be disrupted under pathological conditions, such as radiation, hypoxia, cytokines, hyperlipidemia, and hyperglycemia, which results in the accumulation of ROS, facilitating oxidative stress, cell damage, and diseases, such as cancer, cardiovascular disease, neurological disease, respiratory disease, and so on.^82^ For example, ROS accumulation was observed in cardiovascular diseases like myocardial infarction, heart failure, or other diseases.^83^ In the occurrence of tumors, excessive ROS production promotes tumors by accelerating DNA damage and changes in the tumor microenvironment.^39^

### Oxidative stress changes mitochondrial dynamics

In resemble to ROS production and elimination, the mitochondrial dynamics between fusion and fission are in equilibrium. And dysregulation of such balance will also lead to the occurrence of diseases. In this regard, oxidative stress is one of the factors that alter mitochondrial dynamics balance, mainly in the direction of fission. When ROS increases, the synthesis of mitochondrial fusion-related proteins will be inhibited, thus promoting mitochondrial fission, which in turn may lead to more ROS production and severe mitochondrial dysfunction.^84^ For instance, when the ROS level increased and induced oxidative stress in mouse myocardium, the expression of Mfn1 and Mfn2 decreased, and smaller and fragmented mitochondria appeared in mouse cardiomyocytes, representing that oxidative stress pushed mitochondrial dynamics to the direction of fission.^85^

Changes in mitochondrial dynamics often induce more intense oxidative stress in return. When mitochondrial fission is increased, a greater number of mitochondria are produced, leading to increased ROS production and oxidative stress, which may be a predisposing factor for some diseases. Yuan et al. confirmed that nicotine exposure, a key product of cigarettes, triggered oxidative stress in pancreatic stellate cells via induction of the mitochondrial dynamics towards fission as shown by upregulation of Drp1 and smaller, fragmented-shaped mitochondria.^86^ In cardiomyocytes, nicotine induced the same changes, with increased mitochondrial fission and decreased mitochondrial fusion, which not only induced excessive ROS, but also led to cell apoptosis due to excessive fission.^87^ But at the same time, mitochondrial fission also means that more mitochondria can be produced to provide more energy, which plays an important role in cell proliferation and regeneration.^88^

Interestingly, inducing mitochondrial fusion to inhibit excessive ROS production may protect cells from damage or even death. Liu et al. found that paeonol could mediate the upregulation of OPA1 and promote mitochondrial fusion, which inhibited oxidative stress, improved mitochondrial function, and protected heart function in diabetic cardiomyopathy.^89^ The reason may lie on that paeonol directly targets CK2α, thus inducing the phosphorylation of Jak2-Stat3, which directly binds to the promoter of Opa1 to upregulated Opa1 expression at the transcriptional level.^89^ When mitochondrial fusion is suppressed by the down-regulation of OPA1, resulting in elevated ROS levels while an decrease in antioxidant enzyme levels, finally contributing to oxidative stress and cellular damage.^90^ However, at the same time, activation of mitochondrial fusion may also benefit tumor growth. In the tumor tissues of liver cancer patients, larger mitochondria were observed, accompanied with raised expression of OPA1 and Mfn1, indicating enhanced mitochondrial fusion in tumor tissues, which promoted metabolism and tumor growth.^91^

In general, mitochondrial oxidative stress will lead to the change of mitochondrial homeostasis between fission and fusion, and eventually induce diseases.

### Oxidative stress causes mtDNA damage

mtDNA is mainly located in the mitochondrial matrix, which can be damaged by oxidative stress via either direct or indirect manners. Compared with nuclear DNA, mtDNA is closer to the primary site of ROS production.^92^ Therefore, mtDNA is more susceptible to ROS attack,^93,94^ resulting in various mtDNA damage, such as mtDNA mutation.

On one hand, ROS can directly induce damage to mtDNA damage by oxidative modification of mtDNA bases. Common mtDNA oxidative damage triggered by ROS is 8-oxo-7,8-dihydroguanine (8-oxoG). Guanine has the lowest redox potential among the four bases, making it more susceptible to be damaged from oxidative stress and form 8-oxoG.^95,96^ 8-oxoG can mispair with adenine and therefore leads to G→T mutations during DNA replication.^97^ Although 8-oxoG can be repaired by the 8-oxoguanine DNA glycosylase 1-mediated base excision repair pathway, it continues to accumulate during aging and disease progression.^98–100^

Under oxidative stress conditions, ROS may also interfere with mtDNA replication and repair mechanism. DNA polymerase gamma (POLG), an mtDNA replicase with exonuclease activity, is sensitive to oxidation.^101^ It has been reported that enhanced ROS production in gastric cancer cells resulted in POLG depletion and mtDNA reduction.^102^ In vitro treatment with 1-h H_2_O_2_ demonstrated that the catalytic activity of POLG was reduced to 50% of the original level with significantly lower DNA binding efficiency.^103^ Meanwhile, oxidation was proved to decrease POLG exonuclease activity and DNA mismatch proofreading efficiency, indirectly boosting 20-fold mtDNA mutation than unoxidized one.^104^ Under these conditions, lower ROS levels always along with alleviated mtDNA damage. In an experiment with mouse ovaries, treatment with antioxidant pyrroloquinoline quinone efficiently decreased ROS production, alleviated oxidative stress, relieved mtDNA damage, and increased ATP production.^105^ These findings imply that oxidized POLG causes mtDNA mutation or depletion and in turn results in a variety of diseases.^106,107^

The interplay between oxidative stress and mtDNA damage is bidirectional. On the other hand, oxidative stress leads to mtDNA damage, the damaged mtDNA will exacerbate ROS production, thus aggravating oxidative stress, and forming a vicious cycle. For this reason, lowering mtDNA levels during oxidative stress might be a potential strategy to reduce ROS production and hence control oxidative stress. The transcription factor HIF-1α in mitochondria is an example. Under the induction of oxidative stress, a part of HIF-1α is translocated to mitochondria, where it inhibits the respiratory chain by lowering the expression of mtDNA-encoded mRNA that responses to hypoxia, thus preventing the over-production of ROS and resisting oxidative stress.^71^

### Oxidative stress induces mitophagy

When faced with oxidative stress, activation of mitophagy through various signaling pathways was initiated to remove damaged mitochondria and maintain cell homeostasis. At the same time, the increase of ROS during oxidative stress will lead to mitochondrial membrane potential dissipation, which is another important trigger of mitophagy.^108,109^ The decrease in mitochondrial membrane potential induces PINK1 aggregation and autophosphorylation on the outer mitochondrial membrane, which recruits Parkin to initiate the PINK1-Parkin mitophagy pathway to remove damaged mitochondria.^110–112^ Cadmium, a promotor of excessive ROS production, induces mitophagy through the PINK1/Parkin pathway, while cadmium-induced mitophagy could be reversed after ROS clearance with ROS scavenger.^113,114^

Enhanced mitophagy eliminates damaged mitochondria, which in turn controls ROS and maintains cell homeostasis.^76,115^ On the contrary, damaged mitophagy gives rise to mitochondrial ROS increase and mitochondrial damage, leading to cell damage and even cell death.^116^

## Mitochondria and inflammation

**Fig. 4:**
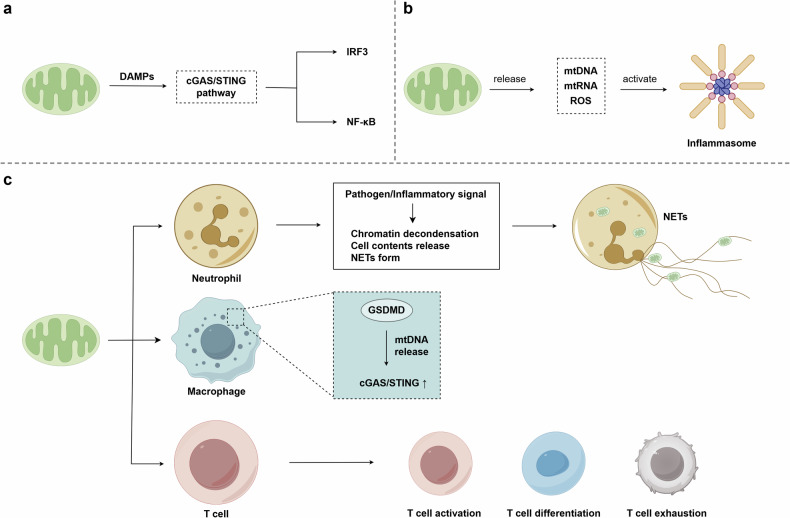
Mitochondrial function on inflammation. **a** mtDNA released from mitochondria into the cytoplasm forms DAMP, which activates the downstream inflammatory signaling via the cGAS-cGAMP-STING pathway. **b** mtDNA, mtRNA, and ROS are all agonists of the inflammasome. **c** Mitochondrial effect on inflammatory cells. ROS promotes the formation of NETs from neutrophils. In macrophages, GSDMD mediates pore formation on the mitochondrial membrane, leading to the release of mtDNA and triggering the DAMP inflammatory pathway. Mitochondria participate in T cell activation, differentiation, and exhaustion through metabolism, oxidative stress, mitochondrial dynamics, and mtDNA damage. DAMP damage-associated molecular patterns, cGAS cyclic GMP-AMP synthase, cGAMP cyclic GMP-AMP, STING stimulator of interferon genes, IRF3 interferon regulatory factor 3, NF-κB nuclear factor-κB, NETs neutrophil extracellular traps, GSDMD gasdermin D. This figure was created using Figdraw

Inflammation is a defense response to the stimulation of inflammatory factors, which usually occurs with pathological changes such as cell necrosis and inflammatory cell infiltration. Many inflammatory cells and cytokines are involved in the inflammatory response. In this process, they may be regulated by mitochondria. Mitochondria can release their contents, forming DAMPs or activating the inflammasomes, which participate in the immunological response. It can also regulate a number of inflammatory cells, including neutrophils, macrophages, and T cells, assisting in responding to injury or infection. (Fig. 4).

### DAMPs

In innate immunity, pattern recognition receptors (PRR) can recognize and bind with ligands on antigens or damaged host cells, inducing immune and inflammatory responses.^117^ PRR can be activated by pathogen-associated molecular patterns (PAMP) and DAMP. PAMPs are intrinsic molecular structures located on the surface of pathogens, which are present in bacteria, viruses, fungi and other pathogens.^118^ Thus the host can recognize the pathogens through PAMP. DAMPs, endogenous molecules including DNA, RNA, and proteins, are produced in response to cellular injury, death or stress.^119,120^ These molecules are usually not recognized as DAMPs when the organism is in a steady state. When the body encounters injury, radiation, infection, drug or shock, DAMPs are released from damaged organelles into the cytoplasm,^121^ and then recognized by PRRs, leading to immune cell activation, the release of cytokines, and other inflammatory responses.^120^

**Fig. 5:**
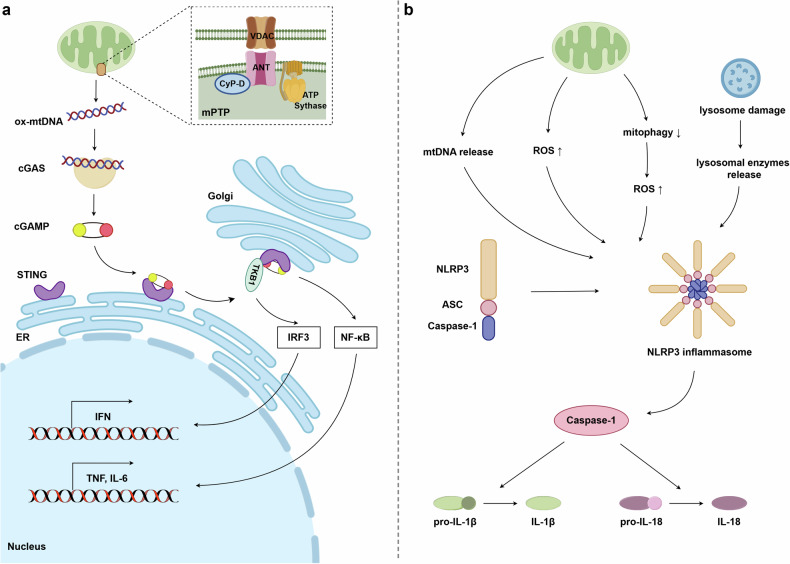
A schematic diagram of mitochondria-induced DAMPs and inflammasomes. **a** Ox-mtDNA is released from mitochondria into the cytoplasm, where it binds to cGAS, catalyzing cGAMP and STING. Then STING translocates from the endoplasmic reticulum to the Golgi apparatus and activates NF-κB and IRF3. IRF3 promotes the transcription of IFN, while NF-κB enhances the transcription of TNF and IL-6. **b** Mitochondrial release of mtDNA, or increased ROS production, activates NLRP3 inflammasome, which activates caspase-1, which processes and matures IL-1β and IL-18. Ox-mtDNA oxidized mtDNA, cGAS cyclic GMP-AMP synthase, cGAMP cyclic GMP-AMP, STING stimulator of interferon genes, TBK1 tank-binding kinase 1, IRF3 interferon regulatory factor 3, NF-κB nuclear factor-κB, IFN interferon, TNF tumor necrosis factor, IL-6 interleukin-6, IL-1β interleukin-1 beta, IL-18 interleukin-18. This figure was created using Figdraw

Many components and products in mitochondria can act as DAMPs, including mtDNA, mtRNA, ROS, and ATP.^17^ Among them, mtDNA is the most widely studied (Fig. 5a).^122–124^ During homeostasis, mtDNA is localized inside mitochondria. When injured or under stress, mtDNA escapes into the cytoplasm with the help of channels in the mitochondrial membrane. Mitochondrial permeability transition pores (mPTP), a nonspecific channel located in the mitochondrial membrane, attribute to the increased permeability of the mitochondrial inner membrane after opening.^125,126^ Yu et al. found that in amyotrophic lateral sclerosis, the 43 kDa TAR DNA-binding protein, a marker protein, can lead to mPTP opening and mtDNA release into mitochondria.^127^ Intriguingly, the voltage-dependent anion channel (VDAC) is a channel in the outer mitochondrial membrane.^128,129^ Xian et al. mentioned that oxidized mtDNA would be cleaved into segments and subsequently transported to the cytoplasm in two steps.^130^ In the first step, these mtDNA fragments were firstly released from the mitochondrial matrix via mPTP in the inner mitochondrial membrane, and then the outer mitochondrial membrane by VDAC.^130^

The translocation of mtDNA from mitochondria to cytoplasm triggers the activation of the cyclic GMP–AMP (cGAMP) synthase (cGAS)–stimulator of interferon genes (STING) signaling pathway. cGAS is a DNA sensor that can recognize and bind with cytosolic DNA.^131^ The combination of cGAS and mtDNA can catalyze cGAMP.^132^ cGAMP is a second messenger, which triggers the activation of adapter STING to form a dimeric configuration.^133–137^ Once activated, STING is transported from the endoplasmic reticulum to the Golgi apparatus.^138,139^ Then, STING recruits tank-binding kinase 1 (TBK1), and inserts the C-terminal tail of STING inserts into TBK1 dimer.^140,141^ TBK1 mediates the activation of nuclear factor-κB (NF-κB) and interferon (IFN) regulatory factor 3 (IRF3).^142,143^ IRF3 is involved in several signal transduction pathways. It is the key factor in the IFN signaling pathway, inducing IFN generation and activation.^144,145^ In the antiviral responses, IRF3 and IFN can limit viral replication.^146,147^ Besides, IFN and IRF3 can cooperate with NF-κB. In antiviral infection, NF-κB and IRF3 have a synergistic effect.^148,149^

Except for mtDNA, other mitochondrial ingredients and production can be DAMPs. mtRNA is the transcription result of mtDNA. The release of mtRNA from mitochondria to cytoplasm also stimulates inflammation and immunity.^150^ The main byproduct in mitochondria, ROS is also included.^151^ Therefore, mitochondria are a vital site to transport DAMPs and induce inflammation.

### Inflammasome

The concept of inflammasome was first proposed by Martinon in 2002 that inflammasome is capable of activating caspase-1 to cleavage pro-IL-1β into IL-1β, which is an inflammatory factor and mediates inflammatory response (Fig. 5b).^152^ Caspase-1 can also activate IL-18 from pro-IL-18. Inflammasome agonists include microbes, ROS, and DNA.^130,153,154^ mtDNA, mtRNA, ROS, and ATP in mitochondria are all able to act as agonists for inflammasomes. In 2011, Nakahira et al. discovered that mtDNA leakage leads to more activation of caspase-1, which is the downstream of the inflammasome.^155^ Leaked mtDNA directly interacts with NLRP3 inflammasome, or indirectly activates the NLRP3 inflammasome via the mtDNA-cGAS-STING pathway.^156–158^ Zhang et al. demonstrated that in nucleus pulposus cells, oxidative stress leads to mPTP opening and cytoplasmic relocation of mtDNA, accompanied by activation of the cGAS-STING axis and NLRP3 inflammasome.^124^ However, mPTP opening inhibitor suppresses the expression of cGAS and STING, thereby inhibiting the activation of NLRP3 inflammasome.^124^ In an acute kidney injury mouse model, Luo et al. observed that the activation of NLRP3 inflammasome and NLRP3 expression were weakened by cGAS inhibitor while STING agonist reversed this inhibition.^159^ Although the mechanism by which cGAS-STING axis activates NLRP3 inflammasome remains unclear, these findings provide new evidence that mtDNA-mediated DAMPs can also mediate inflammasome activation via the cGAS-STING-NLRP3 axis.

In addition to mtDNA, metabolic byproducts of mitochondria also activate the NLRP3 inflammasome. As early as 2009, Shankar had proposed that mitochondria released from necrotic cells activated NLRP3 inflammasome, but the process was inhibited by respiratory chain inhibitors such as rotenone.^160^ Billingham et al. demonstrated that complex I, complex II, and complex III in the respiratory chain all participated in NLRP3 inflammasome activation.^161^

Another important activator of inflammasomes is ROS, the metabolic byproduct of the respiratory chain.^162–164^ There is evidence that ROS engages in inflammasome activation in multiple tissues.^153^ When aggravated ROS production by exogenous stimuli such as lipopolysaccharide is achieved, the activation of inflammasome will be stimulated and lead to cell damage.^165^ Some anti-inflammatory substances, such as neferine, promote ROS clearing by enhancing the activity of SOD, block inflammasome activation, and reduce cell damage.^166^ When mtROS were depleted, NLRP3 inflammasome activation was inhibited.^167,168^ Ren et al. found that ROS depletion not only reduced ox-mtDNA production and the binding to NLRP3, but also prevented NLRP3 deubiquitinating, thereby suppressing NLRP3 inflammasome activation.^169^

ATP is one of the mechanisms that makes mitochondria the activator of inflammasomes. In the necrotic tissue of inflammation, ATP is released extracellularly to form extracellular ATP (eATP), which stimulates purinergic P2X receptor 7, thus activating inflammasome that drives an inflammatory response.^170^ Benefitting from the role of ATP in the activation of inflammatory bodies, one feasible target could be focused on the intervention of ATP in regulating inflammatory response as well as inflammation-related diseases. Metformin effectively decreases the production of ATP and mtDNA, thereby inhibiting NLRP3 inflammasome mitigating acute respiratory distress syndrome and alleviating the resulting lung injury.^171^ In a tumor environment, tumor immune escape mechanisms will clear eATP to avoid inflammasome and inflammation reaction, therefore, CD39 antibody which inhibits ATP transformation to AMP could enhance ATP-mediated activation of inflammasomes and the corresponding inflammatory response.^172^

In contrast, mitophagy serves as a preventative strategy against inflammation via eliminating damaged mitochondria and avoiding the production of excess ROS, hence the change in mitophagy will result in the alternation of inflammasome activation. The PINK1-parkin pathway, as the classical mitophagy pathway, mitigates inflammatory damage through promoting ROS clearing. Inhibition of the PINK1-parkin pathway will increase ROS production and aggravate the activation of inflammasome and apoptosis.^173^ Promoting mitophagy can reduce inflammasomes and thus ameliorate atherosclerosis, colitis, steatohepatitis, etc.^174–176^

Alternatively, when cells are damaged, the function of lysosomes may be affected, leading to lysosomal rupture and the release of a variety of lysosomal enzymes and other molecules. Among them, cathepsin B is thought to bind to NLRP3, thus activating the NLRP3 inflammasome.^177,178^

In conclusion, mitochondria activate NLRP3 inflammasome and initiate inflammatory response through diversified mechanisms including mtDNA leakage, ROS production, ATP, mitophagy and interaction with lysosomes. These mechanisms play key roles in a variety of pathological processes and diseases.

### Inflammatory cells

#### Neutrophil

Neutrophil is an important part of the cellular response to inflammation, which migrates to the inflammatory site and directly engulfs pathogens to kill bacteria.^179,180^ Previous studies have concluded that mitochondria facilitate neutrophil migration in a variety of ways. Mechanisms such as mitochondrial ATP to promote migration signaling, ROS regulation of actin, and mitochondrial calcium regulation to alter neutrophil chemotaxis, are proven to change neutrophil migration.^181^

Importantly, neutrophils can form neutrophil extracellular traps (NETs) to trap and kill pathogens and inhibit infection and inflammation. This mode that neutrophils die with the extrusion of cytoplasmic contents to form NETs, is called NETosis. NET is an extracellular antibacterial mechanism that tends to be produced at the site of inflammation, which can be stimulated by factors like ROS, LPS, interleukin, complement.^182^

The formation of NETs is influenced by mitochondria. ROS is essential for NETosis. During the build of NETs, mitochondria move to the surface of neutrophils and release mtDNA, along with the participation of ROS.^183^ A mitochondria-targeting antioxidant can reduce the production of neutrophil mtROS and result in a decrease in NETosis.^184^ Vorobjeva elaborated on the mechanism of how mtROS promotes NETosis. Formyl-Methionine-Leucine-Phenylalanine can activate the intracellular phospholipase C (PLC)- inositol triphosphate (IP3)-Ca^2+^ pathway. Ca^2+^ can be released from the endoplasmic reticulum. After that, Ca^2+^ unclog the mitochondrial permeability transition pore, and lead to the production and release of mtROS. MtROS are mobilized into the cytoplasm and then activate protein kinase C (PKC), subsequently activating NADPH oxidase and producing more ROS, which together with mtROS induces NETosis.^185^ The exact process by which ROS triggers NETosis is still unclear. Azzouz et al. suggested that DNA damage caused by ROS and subsequent DNA repair might be associated with NETosis.^186^ The subsequent studies found that NETosis could be reduced when the early steps in DNA repair are inhibited.^187^

Since the NETs have been proven to aggravate or even lead to diseases, like stroke, diabetes, and tumors when they over-generate,^188–190^ the inhibition of ROS may prevent disease progression caused by NETs.

#### Macrophages

Mitochondria influence the activation of macrophages in a variety of ways, including mitochondrial metabolism, mitophagy, mitochondrial dynamics, etc. Mitophagy inhibits macrophage activation and thus immune defense, resulting in decreased pathogen clearance and infection, while inhibition of mitophagy in macrophages can promote macrophage activation and thus inhibit infection.^191^

Macrophages could be activated into M1 and M2 macrophages in response to infection, which have different inflammatory regulatory effects.^192^ M1 macrophages secrete pro-inflammatory factors (e.g., IL-6) which play the role of immune defense and promote inflammation, while M2 macrophages produce inhibitory factors like IL-10 to downregulate the immune response. Relevantly, the polarization of macrophages may affect the development of inflammation.^193^ Mitochondrial metabolism may be one key factor affecting macrophage polarization. M1 macrophages are characterized by enhanced aerobic glycolysis, while in M2 macrophages elevated TCA cycle and OXPHOS are observed.^194,195^ In mitochondria, succinate in the TCA cycle is an important factor leading to changes in macrophage metabolism and influences inflammatory signaling. Succinate can promote M2 polarization of macrophages by binding to the succinate receptor 1.^196^ In the tumor environment, succinate could be produced and released by tumor cells, and via interacting with succinate receptor 1 to activate phosphoinositide-3-kinase/protein kinase B pathway and upregulate HIF-1α, inducing macrophage polarization, and enhancing tumor cell metastasis.^197^ Besides, succinate can mediate the progress of inflammation. In the cardiovascular system, succinate released by macrophages stimulated the production of inflammatory cytokine IL-1β by activating HIF-1α, thus inducing endothelial inflammation and then promoting atherosclerosis.^198^

Mitophagy also takes part in the polarization of macrophages. In this regard, taurine can block Pink1-mediated mitophagy to maintain the number of mitochondria and inhibit glycolysis required for the polarization to M1, thus inhibiting the M1macrophages polarization.^199^

Mitochondria also contribute to the inflammatory response of macrophages by ROS. In the inflammatory response, the phagocytosis of macrophages can aggravate the inflammatory response, in which ROS is involved. When macrophages recognize certain foreign substances, they will trigger inflammation and diseases through the inflammatory factor pathway and oxidative stress response. For example, large exposure to asbestos may be one of the factors of pulmonary inflammation, due to some occupational factors. During the progression, asbestos can be recognized and taken up by macrophages, then stimulating the secretion of IL-1 family and the activation of NLRP3 inflammasomes, thus inducing inflammation.^200^

In recent years, the gasdermin D (GSDMD)-mediated mitochondrial damage mechanism in macrophages has received increasing attention. GSDMD is well known as a key point in the process of pyroptosis.^201,202^ Previous studies have found that GSDMD is capable of forming pores in the cell membrane, triggering the release of cellular contents and initiating inflammatory responses.^203,204^ Recently, researchers have further uncovered that GSDMD also mediates mitochondrial damage during the activation of macrophages. In macrophages, NLRP3 inflammasome activates caspase-1.^205–207^ GSDMD is the substrate of caspase-1, which will be cleaved to separate GSDMD-N from GSDMD-C.^208–210^ GSDMD-N binds to membrane lipids, such as cardiolipin and phosphoinositide, and crosslinks to oligomers, thereby forming membrane pores with inner diameters ranging from 10 to 14 nm.^211,212^ When GSDMD induced pore formation in the mitochondrial membrane, one of the consequences is the escape of mtDNA to form DAMPs.^213,214^ This would trigger the subsequent mtDNA-cGAS-STING pathway, activating the downstream inflammatory response.^213^ Another consequence of GSDMD-N forming mitochondrial pores is the destruction of mitochondrial membrane integrity and loss of mitochondrial membrane potential.^215^ In conclusion, mitochondrial membrane pore formation and changes in membrane potential drive ROS production and inflammation.

#### T cell

Mitochondria play a critical role in T cell activation and differentiation. When stimulated by a foreign antigen, T cells initiate an immune program in response to the antigen attack. Primitive T cells differentiate into effector T cells, cooperating with other immune cells to resist the attack of foreign antigens, and can also form memory T cells to prepare for the next antigen attack in advance. Mitochondria undergo dynamic changes in the process of T cell differentiation. Effector T cells are characterized by small, scattered mitochondria, whereas mitochondria in memory T cells are fused, which may suggest that mitochondria are characterized by fission in effector T cells and tend to fusion in memory T cells.^216^ The fusion of mitochondria was affected by OPA1, which can change mitochondrial inner membrane fusion. When T_H_17 cells were deleted with OPA1, the fusion of the mitochondrial membrane was impaired, and the expression of inflammatory factor IL-17A was influenced and then decreased.^217^

Aside from mitochondrial dynamics, the products produced in mitochondria can also affect T-cell differentiation. Sirtuin3 (sirt3) is a deacetylase located in the mitochondrial matrix, participating in the SUMO-specific protease (SENP1)-Sirt3 axis to regulate the metabolic activity of mitochondria together with its upstream protein SENP1. In the process of memory T cell development, low glucose could activate the SENP1-Sirt3 axis, thus enhancing oxidative phosphorylation, and promoting the survival of memory T cells.^218^

Mitochondria also influence T cell fate. When T cells are continuously stimulated, mitochondrial function is inhibited, impairing TCA cycling and oxidative phosphorylation. This restriction in ATP synthesis leads to ROS accumulation and oxidative stress, which in turn inhibits T cell self-renewal and contributes to T cell exhaustion.^219^ During TIL exhaustion, changes in mitochondrial dynamics, such as mitochondrial membrane structure destruction and cristae length change, are commonly observed.^220^ An increase in mitochondrial mass and hyperpolarized mitochondria result in mtDNA damage, which affects T cell senescence.^221^

## Mitochondria and aging

Aging is the result of multiple confounding factors, which is not only a naturally decline process of the human body but also can be induced by other factors such as diseases. Mitochondrial dysfunction is one of the hallmarks of aging, and its interaction with oxidative stress and inflammation also contributes to the regulation of aging, given that oxidative stress and inflammation are also triggers of aging. In this section, we will discuss how mitochondria act as a central hub in linking oxidative stress, inflammation, and aging.

### Mitochondrial dysfunction in senescent cells

There are numerous theories about aging, but aging remains a complex process influenced by various factors.^222^ Currently, researchers have indicated that the aging process is accompanied by changes in mitochondria, including the increase of mtDNA mutations, changes in mitochondrial metabolism and mitophagy, the breakdown of mitochondrial dynamic balance, etc.^223–226^ When mitochondrial dysfunction occurs, it often shows a decline in physical function and the occurrence of age-related diseases.^227–229^

#### Mitochondrial dynamics

According to the free radical theory, aging is caused by the accumulation of free radicals in cells.^230^ As mentioned earlier, ROS accumulation and associated oxidative stress are important causes of mitochondrial dynamics alterations. However, the regulatory role of mitochondrial dynamics in aging has not yet been clearly explained. Many mechanisms are involved in the changes of mitochondrial dynamics and aging. On the one hand, in some senescent cells usually exist large and long mitochondria.^231^ However, on the other hand, some studies found enhanced mitochondrial fission in specific aging contexts.^232^ During aging, intracellular calcium regulation capacity declines, calcium homeostasis is unbalanced. Calcium dysregulation affects mitochondrial dynamics-related proteins.^233,234^ Increased intracellular calcium concentration has been observed in a variety of senescent cells.^235,236^ Calcium/calmodulin-dependent protein kinase can be activated by high calcium concentration, and then phosphorylates Serine616 on Drp1.^237,238^ Phosphorylated Drp1 translocate to the mitochondria and enhance mitochondrial fission.^239^ In addition, high concentrations of calcium ions also indirectly down-regulated Mfn1 and Mfn2, further inhibited mitochondrial fusion, that ultimately promoted mitochondrial fission in senescent cells.^237^

Alternatively, alterations in signaling pathways in senescent cells also reprogram mitochondrial dynamics. AMP-activated protein kinase (AMPK) signaling pathway activity decreases with aging.^240,241^ This is associated with decreased energy metabolism, oxidative stress, and inflammation. Inhibition of AMPK reduced its inhibitory effect on Drp1, disturbing mitochondrial dynamics.^242^

In addition to the factors mentioned above, other elements also contribute to the imbalance of mitochondrial dynamics in aging. For example, radiation has been shown to induce skin aging, leading to oxidative stress and cellular damage. In human epidermal keratinocytes, ultraviolet radiation B (100–500 mJ/cm²) induces mitochondrial fragmentation, and the inhibition of Drp1 mitigates this radiation-induced fission. Therefore, radiation may enhance Drp1-mediated mitochondrial fission, which is associated with skin aging.^243–245^

Such imbalance in dynamics leads to the accumulation of dysfunctional mitochondria. Superfluous damaged mitochondria further lead to ROS overproduction, affect mitophagy due to overburdened workload, and finally aggravate the decline of cell function.

#### Energy metabolism

During aging, a series of changes occur in mitochondrial energy metabolism, including decreased ATP production, increased ROS, and imbalance of mitochondrial homeostasis. Mitochondrial energy metabolism disorders not only affect cell function, but also cause systemic metabolic abnormalities, and inducing age-related diseases. Diseases like neurodegenerative disorders, metabolic syndromes, and cardiovascular diseases, all of which are linked to mitochondrial dysfunction.^246–248^

Aging affects the efficiency of ATP production, with ROS accumulation. With aging, mitochondrial electron transport chain function gradually diminishes, leading to a decrease in ATP production efficiency.^249^ It has long been known that the activity of complexes I and II in the electron transport chain decreases with aging.^250–252^ In addition, aging affects the mitochondrial membrane potential and proton gradient across the mitochondrial inner membrane, which is the driving force for ATP synthesis.^253^ In senescent cells, mitochondrial proton leak was observed, associated with the weakened proton gradient and further reducing the efficiency of ATP synthesis.^254,255^

Recent studies have shown improving mitochondrial energy metabolism disorders may delay aging. Small extracellular vesicles extracted in young plasma have been found to optimize mitochondrial energy metabolism, and then trigger the shift in aging-associated functional deterioration.^256^ Besides, extracting small extracellular vesicles from young mice and injecting them into aged mice can significantly prolong the lifespan of the latter.^256^ Accordingly, it is interesting to wonder whether the transfer of sole mitochondria has the ability to exert such a lifespan prolong effect, especially considering that mitochondria transfer has been validated as an effective strategy to combat diseases.^257^

Aging causes ROS accumulation.^258,259^ In senescent cells, the mitochondrial fission increases, causing the increase of dysfunctional mitochondria. Dysfunctional mitochondria bring about less respiration and more ROS production. Meanwhile, in aging mice, the activity of catalase and SOD reduced a lot, which meant the incline of antioxidation ability.^260^ This imbalance of oxidation and antioxidation will further aggravate the accumulation of ROS. Consequently, the persistent buildup of ROS can damage cellular components, contributing to the progression of age-related diseases. Ultimately, restoring mitochondrial function and enhancing antioxidant defenses may be critical strategies for mitigating the effects of aging.

In addition, one of the recent focuses is the NAD^+^-SIRT3 axis.^261,262^ Usually, mitochondrial dysfunction impairs NAD⁺ regeneration from NADH. The dysregulated NAD^+^/NADH ratio, together with the absence of SIRT3, may cause cell cycle and mitosis arrest, and mediate metabolic changes in aging by activating AMPK to initiate senescence via the classical p53 pathway.^263^ Under hyperglycemic conditions, overexpression of mitochondrial uncoupling protein 2 induced the up-regulation of NAD^+^ and SIRT3, and alleviated senescence caused by hyperglycemia.^264^

#### mtDNA mutation

In response to mtDNA damage, several mtDNA repair pathways have been reported to fix these broken nucleic acids. The deterioration of the quality and quantity of mtDNA is significantly important to mitochondrial dysfunction during aging.^265–267^ There are various evidences that mtDNA mutations accumulate continuously as age increases.^268,269^ Recently, single-cell sequencing also confirmed the presence of a large number of mtDNA mutations in senescent cells, and more than 60% of the mutations occurred in protein-coding genes.^270^ mtDNA mutations may originate from ROS attacks, accumulated replication errors, and environmental factors such as radiation and chemicals. These mutations store up and lead to abnormal mitochondrial metabolism and function, triggering apoptosis.

mtDNA mutations have long been recognized as the driving force of aging.^271,272^ Mutations of mtDNA which encodes respiratory chain proteins affect the respiratory chain stability and result in premature aging.^273^ During the process of aging, unrepaired mtDNA mutations accumulate, eventually leading to mitochondrial dysfunction and diseases.^274^ Smith et al. demonstrated that age-related somatic mtDNA mutations could lead to defects in OXPHOS function, alter metabolic patterns, and accelerate the development of intestinal cancer.^275^ For example, more mtDNA mutations were observed in aged oocytes than in young ones, and the increased mutations inhibited the ovarian function, resulting in fewer available follicles and affecting female fertility.^276^

Understanding the link between mtDNA mutations and aging could pave the way for novel approaches to delay aging and treat related diseases.

#### Mitophagy

As aging progresses, the efficiency of mitophagy gradually declines.^277–279^ When dysfunctional mitochondria cannot be cleared in time by mitophagy, damaged mitochondria accumulate, which results in insufficient energy supply as well as overproduced ROS, thereby triggering intense oxidative stress and cellular damage. Especially the subsequently increased mitochondrial fission during aging exacerbates this consequence.

Given the crucial role of mitophagy in maintaining mitochondrial function, researchers are exploring methods to enhance mitophagy as a potential strategy to delay aging and treat related diseases. There is evidence that Parkin-mediated mitophagy alleviates cardiac aging.^280^ Shank3 protein is a negative regulator of Parkin-mediated mitophagy and is enriched in the heart with increasing aging.^281^ Shank3 knockout can restore mitophagy, inhibit mitochondrial ROS production, and improve cardiac function in aging mice.^281^ Similarly, in germ cells, enhanced mitophagy restores the function of senescent cells. In oocytes from aged mice, enhancing Parkin-mediated mitophagy by supplementing spermidine improved oocyte fertility.^282^ Besides, improving BNIP3-mediated mitophagy had the same effect. In *Drosophila*, enhancing BNIP3-mediated mitophagy in the nervous system improves mitochondrial homeostasis, thereby extending lifespan, and improving muscle and gut homeostasis.^283^

### Mitochondria in aging and oxidative stress

Harman proposed the free radical theory of aging in the 1950s, which hypothesized that the increase of free radicals, especially oxygen free radicals, would induce oxidative stress, causing damage to a variety of bioactive substances and accelerating aging.^230^ Since then, more attention has been paid to the effect of oxidative stress on aging.^284,285^ In 2014, Kovalenko et al. studied the ROS content in neutrophils of different age groups and found that the intracellular ROS content increased significantly in people aged 60–89 years compared with those aged 20–59 years.^286^ This indicates that aging is accompanied by ROS accumulation, which is produced by the imbalance of oxidation and antioxidation. During aging, mitochondrial functions decline, accompanied with more ROS production and an imbalance between oxidation and antioxidation. Marzani et al. found that the GSH/GSSG ratio in the rectus abdominis of the elderly decreased significantly when comparing the antioxidant levels in muscles between the elderly and the young, which indicated that antioxidant capacity changed with aging.^287^ These findings underscore the relationship of oxidative stress with aging process.

Mitochondrial dysfunction is associated with a wide range of diseases, such as neurodegenerative diseases, diabetes, age-related macular degeneration, and cancer.^288–290^ As mentioned above, oxidative stress accompanied by mitochondrial dysfunction can damage the function of mitochondrial OXPHOS, induce mtDNA damage, and change mitophagy. Damaged mitochondria, in return, aggravate oxidative stress. This vicious cycle will lead to an intensification of ROS accumulation, which ultimately leads to the progression of aging. Consequently, mitochondria are of great importance for connecting oxidative stress and aging, making them a key target for therapeutic interventions aimed at mitigating age-related diseases.

### Mitochondria in aging and inflammation

It has been demonstrated that inflammatory responses effectively promote aging.^291,292^ The activation of inflammation leads to cell damage, and eventually, the accumulation of damage accelerates aging. Several studies suggest that the core of aging is persistent chronic inflammation.^293^ A study in mice demonstrated that the concentration of IL-1α and TNF-α in the serum of 52-week-old mice was significantly higher than that of 12-week-old mice.^294^ Similarly, IL-6 levels in the aortas of aged mice were also higher than that in young mice.^295^ Many inflammatory responses not only promote aging but are also associated with aging-related diseases. Li et al. compared macrophages in colorectal cancer tissues of elderly and young patients and found that more macrophages infiltrated in the tumor tissues of elderly patients.^296^ In addition, in the further study with tumor-bearing mice models, macrophages from mice of different ages exist diverse phenotypes, indicating that aging is accompanied with changes in inflammatory cells and will affect tumor progression.^296^

It has been demonstrated that mitochondria induce inflammatory responses via releasing DAMPs, stimulating inflammasomes, and activating inflammatory cells. These mitochondria-related inflammatory mechanisms establish a link between aging and inflammation.

Aging induces inflammatory responses by affecting mtDNA leakage in a variety of ways. Zhong et al. found that in senescent macrophages, mtDNA leakage promoted the cGAS-STING inflammatory pathway, and decreased PINK1/Parkin mediated mitophagy.^297^ However, overexpression of PINK1 inhibit the mtDNA-cGAS-STING pathway, so impaired mitophagy boosts inflammatory responses by promoting mtDNA DAMPs in aging.^297^ Subsequently, Victorelli et al. showed in senescent cells widespread mitochondrial outer membrane permeabilization in part of mitochondria enhanced mtDNA leakage to the cytoplasm and subsequent activation of the cGAS-STING pathway.^298^ And mtDNA leakage can be prevented by inhibiting mitochondrial outer membrane permeability, thus improving the frailty of aging mice, and prolonging the mice life span.^298^ Additionally, promoting mitophagy also helps mitigate the inflammatory response in aging. Jimenez-Loygorri et al. constructed a mito-QC reporter mouse to evaluated mitophagy level and found that mitophagy became either stable or increased in tissues such as kidney, brain and retinal pigment epithelium of physiologically aging mice. And more mtDNA-induced cGAS-STING inflammatory response was observed in aged mice.^299^ After using the mitophagy inducer urolithin A, increased mitophagy significantly down-regulated the mtDNA mediated cGAS-STING axis, thereby alleviating the inflammatory response caused by aging.^299^

Many studies have demonstrated that mitochondrial-activated inflammasomes induce aging.^300^ In senescent cardiomyocytes, increased ROS production and subsequent activation of NLRP3 inflammasomes are observed.^301^ Besides, in NLRP3-deficient mice, the aging-related metabolic pathways was changed, together with prolongated telomeres, indicating that the inhibition of inflammasomes may alleviate aging.^302^ In addition, other inflammatory responses mediated by mitochondria, such as the activation of inflammatory cells, also promote aging. T cells are not considered to be conventional proinflammatory cells. However, T-cell dysfunction may speed up aging. Desdin-Mico et al. demonstrated that in T cells with deficient mitochondrial transcription factor A genes (*Tfam*), mtDNA content decreased, leading to mitochondrial dysfunction.^303^ In such mice containing *Tfam*-deficient T cells, inflammatory factors like IL-6 and Stat-1 were activated prematurely, thus stimulating aging-associated inflammatory responses and premature aging.^303^

## Mitochondrial dysfunction and diseases

**Fig. 6:**
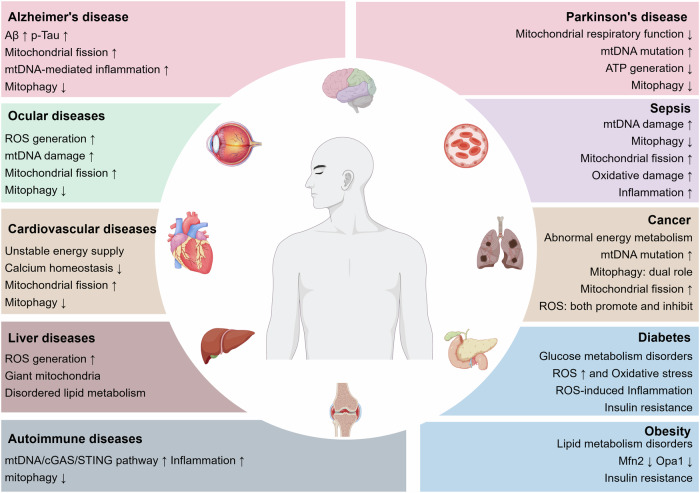
Mitochondrial dysfunction in diseases. Mitochondrial dysfunction is widely observed in a variety of diseases, including cancers, cardiovascular diseases, neurodegenerative diseases, metabolic disorders, liver diseases, autoimmune diseases, and ocular diseases. This figure was created using Figdraw

Note that the role of mitochondria in oxidative stress, inflammation, and aging has been discussed, it is important to recognize how mitochondrial dysfunction contributes to disease through the mechanisms outlined above. Many diseases, such as cancers, cardiovascular diseases, neurodegenerative diseases, metabolic disorders, liver diseases, autoimmune diseases, and ocular diseases, are closely associated with mitochondrial dysfunction (Fig. 6). The onset of these diseases is often accompanied by abnormalities in mitochondrial energy metabolism, increased oxidative stress, and exacerbated inflammatory responses, further aggravating the pathological processes.

### Cancers

It is well-established that mitochondria dysregulation is involved in tumor initiation, progression and metastasis.^304–306^

In 1956, Warburg proposed a phenomenon known as the “Warburg effect”, that tumor cells consume large amounts of glucose to produce energy through aerobic glycolysis.^307^ However, more and more researches demonstrated that ROS promotes and inhibits tumor development and metastasis.^39^ On the one hand, ROS facilitates tumor development by inducing mutations in some genes by oxidizing DNA. This process activates oncogenes or inhibits tumor suppressor genes, thereby promoting tumor formation and development. Moreover, ROS serves as signaling molecules that enhance cellular proliferation and survival pathways. They activate key signaling cascades such as MAPK, PI3K/Akt, and NF-κB, which are crucial for cell growth and survival.^308,309^ So ROS-induced signaling promotes the proliferation of cancer cells and contributes to tumor progression. Moreover, ROS facilitates tumor invasion and metastasis by enhancing the migration of cancer cells, often through the regulation of matrix metalloproteinases and epithelial-mesenchymal transition. On the other hand, ROS may have an inhibitory effect on tumor development and metastasis. ROS can inhibit tumor development due to its cell toxicity. When the level of ROS is too high, it induces severe oxidative stress in cells, leading to DNA, protein or lipid damage in cells, and causing cell apoptosis or necrosis. In tumors, this high level of ROS inhibits tumor growth by inducing cell death or autophagy, which opens up the idea of a therapeutic approach aimed at elevating ROS levels. For example, certain anti-cancer therapies like radiotherapy and some chemotherapy drugs could produce oxidative stress in tumor cells by increasing ROS components and induce tumor cell death.^310^ In addition, under the tumor microenvironment, ROS plays a crucial role in affecting cancer cells as well as stromal and immune cells.^311^ It is noteworthy that elevated ROS levels will promote angiogenesis, immune evasion, and even drug resistance, which act as a supportive niche for tumor growth. In summary, ROS serves as a double-edged sword in cancer.^312^ Understanding the intricate balance of ROS in tumor biology is key to developing targeted therapies that can modulate ROS levels to inhibit tumor growth effectively.

Mutation of mtDNA is commonly observed in many types of cancers.^313,314^ These mtDNA mutation covers almost every complex of OXPHOS. In brain gliomas patients, mutations of mtDNA, including Cytochrome b (CYB) gene, ATP6 gene and cytochrome oxidase subunit I (CO1) gene are observed.^315^ In prostate cancer, CO1 mutation occurs more in cancer patients, especially mutation of conserved amino acids.^316^ Mutations in OXPHOS mtDNA lead to the inhibition of the respiratory chain, which can then increase the production of ROS and further enhance the mtDNA damage. This forms a vicious circle between ROS and tumor growth.

Mitophagy is also a double-edged sword for tumorigenesis.^317^ Mitophagy can suppress tumors due to its role in mitochondrial homeostasis. Mitophagy-related proteins Parkin, BNIP3, and NIX are all considered to have tumor-suppressive functions.^318–320^ When mitophagy is inhibited, mitochondrial homeostasis is easy to be dysregulated, and inappropriate mitochondrial function and excessive ROS appear.^321^

Sometimes, mitophagy may promote tumors. Increased mitophagy always along with reduced mitochondrial damage as well as lower ROS levels, promote cell survival. Namely, mitophagy will benefit tumors from evading chemotherapy by alleviating oxidative stress and clear damaged mitochondria.^322^ Methyltransferase-like 3 (METTL3) induces PINK1-Parkin mitophagy by promoting the methylation of decapping protein 2, enabling small cell lung cancer resistant to chemotherapy.^323^

Mitochondrial dynamics is lopsided in tumors, as mitochondrial fission promotes tumor development.^324–327^ Mitochondrial fission-related genes, such as Drp1, are upregulated in several types of tumors, associated with poor prognosis and metastasis.^328^ Fragmented and smaller mitochondria were observed in metastatic breast cancer cells.^329^ In hepatocellular carcinoma, increased mitochondrial fission promotes pseudopodia formation and induces metastasis of tumor cells.^330^ Via targeting inhibit DRP1, the incidence of brain metastases was effectively reduced in breast cancer.^329^ Therefore, mitochondrial fission level concerns the metastasis of cancers, and the inhibiting of mitochondrial fission has the potential to be a promising target for better prognosis.

### Cardiovascular diseases

The heart is an energy-intensive organ. Mitochondria provide energy for cardiomyocytes through OXPHOS and regulate lipid metabolism as well as calcium homeostasis in cardiomyocytes. It has been widely reported that mitochondria module the myocardial cells and vascular muscle cells.^331,332^ When mitochondria disorders occur, cardiac energy supply, calcium homeostasis, oxidative stress, as well as cell fate will all be affected, leading to changes in cardiac structure and function. Therefore, mitochondrial dysfunction is involved in many cardiovascular diseases, such as heart failure and myocardial infarction.^27,28,333^

In the process of mitochondrial dysfunction, imbalanced ROS production and subsequent oxidative stress play an important role, leading to cardiac injury. The accumulation of ROS influences calcium homeostasis in cardiomyocytes. ROS causes cardiomyocyte electrophysiological dysfunction by regulating calcium channels.^334^ ROS affects cardiac contraction by regulating sarcoplasmic reticulum Ca^2^^+^-ATPase.^334^ Recently, Seok et al. demonstrated that oxidative modification by ROS formed 8-oxoguanine in miRNAs seed regions, which led to adenine mispairing mutation with mRNA, and promoted cardiac hypertrophy.^335^

In cardiovascular diseases, mitochondrial dynamics homeostasis is unbalanced, often leading to increased mitochondrial fission.^248^ Drp1 is a fission-promoting protein. In many cardiovascular diseases appear inappropriate mitochondrial fission and Drp1 upregulation, such as heart failure, myocardial infarction and cardiac hypertrophy.^336^ For example, in mice with myocardial ischemia-reperfusion, Drp1-mediated mitochondrial fission is upregulated, while Drp1 inhibitor can down-regulate mitochondrial fission, increase ATP, and improve cardiac function.^337^ Yan et al. found that DEAD-box helicase 17 acted together with B-cell lymphoma 6 to inhibit Drp1 in cardiomyocytes to regulate cardiomyocyte homeostasis.^338^ However, in heart failure, DEAD-box helicase 17 and B-cell lymphoma 6 are downregulated, which would reverse the inhibition of Drp1 and promote mitochondrial fission.^338^

Mitochondrial fission generates fragmented mitochondria that recruit Parkin-mediated mitophagy and maintain cardiomyocyte homeostasis.^336^ In various cardiovascular diseases, including cardiomyopathy, heart failure, and myocarditis, stable mitophagy is essential for maintaining cardiac function.^339–341^ During reperfusion after myocardial infarction, downregulation of zinc transporter ZIP7 increases mitochondrial zinc ions, leading to mitochondrial depolarization and up-regulation of the PINK1-Parkin pathway, which enhanced mitophagy and reduced infarct size.^342^ However, the damaged mitophagy leads to ROS accumulation, inflammasome activation, and lipid accumulation, which results in cell damage.^343^ In inflammatory injury, mitophagy regulates cardiomyocyte injury by restricting inflammasome hyperactivation.^344^ Yu et al. revealed the dynamic change of mitophagy and cardiomyocyte inflammation.^345^ In the early stage of cardiomyocyte inflammatory injury, mitophagy is induced to clear the injury, while as inflammation expands, mitophagy may be inhibited.^345^ This suggests that mitophagy works as a potential target for inflammatory heart disease.

### Neurodegenerative diseases

Mitochondrial dysfunction in Alzheimer’s disease (AD) includes mitochondrial dynamic changes, a decrease in ATP generation, increased oxidative stress, mitophagy damage and mtDNA mutation.^23,346,347^ The hallmark changes of AD are an accumulation of amyloid beta (Aβ) protein and phosphorylated Tau (p-Tau) protein.^348–351^ Their over-accumulation may result in abnormal mitochondrial dynamics and mitophagy.^246,352,353^

In the nervous system of AD patients, mitochondrial fission is more universal.^288^ Manczak et al. identified that in AD patients, Drp1 and Fis1 were up-regulated, while Opa1, Mfn1 and Mfn2 was down-regulated, and double-labeled immunofluorescence showed that Drp1 was co-localized with and Aβ in AD brains, indicating the interaction between Aβ and Drp1.^354^ In their subsequent study, they further demonstrated that Drp1 also interacted with p-tau in the brain of AD patients.^355^ The interaction of tau and Aβ with Drp1 is responsible for the increased mitochondrial fission and abnormal mitochondrial dynamics in the brains of AD patients, which are involved in neuronal damage. However, other studies have come to a different conclusion. DuBoff et al. found mitochondria elongation in Drosophila and mouse neurons.^356^ Since Tau can bind and stabilize actin, when Tau expression was increased, the over-stabilized actin prevented Drp1 from localization to mitochondria, down-regulated mitochondrial fission, lengthened mitochondrial morphology in neurons, and overproduced ROS, which induced neuropathy.^356^

In the brain sample of AD patients, mitophagy levels decreased 30–50% compared with the normal ones, at the meantime downregulated PINK1 and BNIP3L were observed.^357^ Accumulation of Aβ and Tau impair PINK1-Parkin mediated mitophagy in AD patients. Tau was reported to damage mitophagy by preventing Parkin translocating to mitochondria.^358^ In AD model, an increase of Tau and Aβ protein precursor inhibited Parkin translocation to mitochondria, thus Parkin was unable to label impaired mitochondria, and mitophagy was damaged.^359^ Therefore, enhanced mitophagy is regarded as a strategy to alleviate neurodegeneration^360,361^ by upregulating PINK1 and Parkin in neurons, thus increasing the activity of microglia to phagocytose Aβ plaques, and improving cognitive and memory function.^357^

Apart from this, Aβ mediate neuroinflammation of AD through mtDNA-mediated inflammation. In AD, Aβ induces neutrophil infiltration into the central nervous system through the mtDNA-STING-NLRP3/IL-1β axis, which may lead to neuronal damage.^362^

Parkinson’s disease (PD) is another age-related neurodegenerative disease characterized by the degeneration of dopaminergic neurons in the substantia nigra.^363^ In PD patients, mitochondrial dysfunction, including respiratory hypofunction, mtDNA mutation, and decreased mitophagy were found to be involved in the pathogenesis of PD. Earlier studies focusing on post-mortem brains from PD patients verified the decline of OXPHOS, especially complex I activity.^364,365^ Besides, aberrant mitophagy triggered by PINK and Parkin mutations are associated with PD development in familial PD patients.^366^ In conclusion, mitochondrial dysfunction is involved in the development of PD by affecting its pathological state, inspiring that the intervention of mitochondrial dysfunction may work as a potential strategy for future PD treatment.

### Metabolic disorders

Metabolic disorders are a group of diseases that change the normal metabolic process, which can affect the function of sugars, fats, proteins, enzymes in the body, thus affecting metabolism. Common metabolic disorders include diabetes, phenylketonuria, obesity, and Wilson’s disease.^367^

Diabetes is a kind of metabolic disorder disease characterized by hyperglycemia, whose disorder contains glucose metabolism disorders, lipid metabolism disorders, microangiopathy, neuropathy, etc. Mitochondrial dysfunction is important in diabetes. ROS has been reported to be elevated in adipose tissue, liver, and muscle in diabetic patients.^247^ Hyperglycemia is the motivation of excessive ROS and oxidative stress, while activating the inflammasome and exacerbating inflammation.^368^

Oxidative stress is associated with insulin resistance.^369^ Advanced glycation end products, which are generated by glucose in a non-enzymatic manner, also promote ROS production and endoplasmic reticulum stress, and then induce insulin suppression via the protein kinase RNA-like endoplasmic reticulum kinase/forkhead box O1 pathway.^370^ Down-regulation of the mitochondrial fusion protein Mfn2 in the muscle and liver of diabetic patients also induces oxidative stress and leads to insulin resistance.^371,372^

Oxidative stress and inflammation induced by elevated ROS are involved in many diabetic complications. In the vascular complications of diabetes, ROS accumulation, mitochondrial dynamics changes, mitophagy, and their induced inflammation all lead to vascular endothelial damage in diabetes.^373–375^ Besides, ROS excessive production and activated inflammasomes result in delayed wound healing in diabetic patients, and enhancing antioxidation to reduce ROS can accelerate wound healing.^376,377^

Obesity is a metabolic disorder caused by genetic, environmental, and other factors, which is related to a variety of metabolic diseases, such as diabetes, hypertension, cardiovascular, and cerebrovascular diseases. In obese patients, there are also disturbances in glucose metabolism, lipid metabolism, and insulin resistance.^378,379^ These metabolic abnormalities contribute to mitochondrial dysfunction. Mice with high-fat diet showed downregulation of Mfn2 and Opa1 in multiple organs.^380^ Mfn2 interacts directly with perilipin 1 to promote adrenergic and stimulate interaction between mitochondria and lipid droplets, affecting lipid metabolic processes and thermogenic function in brown adipose tissue.^381^ Therefore, mitochondrial dynamics is involved in the regulation of obesity by altering lipid metabolism.

### Sepsis

Sepsis is a severe inflammatory response syndrome triggered by the invasion of pathogens into the human body.^382^ According to the pathogenesis, it activates an excessively intensive immune response, leading to the release of a large amount of inflammatory factors, cell damage, and multiple organs dysfunction.^383^ When sepsis keeps progressing, it may induce septic shock, multiple organ failure, and even death.

There is much evidence for mitochondrial dysfunction and consequent oxidative stress and inflammation in sepsis. An observational study illustrated that patients with sepsis exhibit decreased respiratory levels compared with the controls, especially for respiratory levels in complex I.^384^ Similar conditions were obtained in sepsis children, besides the decreased respiratory capacity, the mtDNA/nDNA ratio gradually increased as sepsis progressed over time.^385^ Mitochondrial respiratory dysfunction, resulting in ATP reduction and energy metabolism disorders, will further affect normal cell functions, leading to organ dysfunction.

As sepsis spreads and aggravates, manifestations of mitochondrial dysfunction will occur in several organs.^386,387^ Several studies have shown that mitochondrial dysfunction is involved in sepsis-induced multiple organ failure.^388^ The common mitochondrial characteristics of sepsis-mediated multiple organ failure are mtDNA damage, oxidative damage, down-regulation of mitophagy, and increased mitochondrial fission, which appeared in kidney injury, myocardial injury, and lung injury.^389–391^ Zou et al. demonstrated that improving mitochondrial metabolism, reducing oxidative stress and mitochondrial fission via the deletion of DNA-dependent protein kinase catalytic subunit (DNA-PKcs), thus alleviating sepsis-mediated myocardial injury, liver injury, and kidney injury in a sepsis model.^386^ Subsequently, Ma verified that the inhibition of DNA-PKcs effectively reduced the expression of inflammatory factor INF2 and excessive mitochondrial fission, following with alleviated cardiomyocyte dysfunction.^392^ These results indicated that mitochondrial dysfunction, as well as the induced oxidative stress and inflammation aggravate sepsis and related complications.

### Liver diseases

Liver diseases refer to a series of pathological conditions that affect the normal function of the liver, contributing to a huge global health burden.^393–395^ Common etiologies include infection, metabolic disorders, drugs or toxins, alcohol, genetic factors, and liver tumors.^396–399^ As a key component of energy and metabolism, mitochondria play a crucial role in maintaining liver health by regulating energy production, ROS generation, and apoptosis. Mitochondrial dysfunction is closely related to the occurrence and development of various liver diseases, including non-alcoholic fatty liver disease, viral hepatitis, and hepatocellular carcinoma.^400–402^ In liver diseases like non-alcoholic fatty liver disease (NAFLD) and cirrhosis, mitochondrial dysfunction leads to impaired fatty acid oxidation, excessive ROS production, and oxidative stress. All the above-mentioned issues will contribute to liver inflammation, fibrosis, hepatocyte damage, and so on. Abnormal mitochondrial content and energy metabolism, mitochondrial dynamics changes are all associated with mitochondrial dysfunction in fatty liver diseases.^403^ Moreover, viral infection promotes mitophagy and mitochondrial fission, further inhibiting the electron transport chain and ATP production in viral hepatitis.^404^

Mitochondria are the main site of fatty acid oxidation and are involved in liver diseases, which are tightly associated with lipid metabolism.^400,405^ An inhibition in mitochondrial fusion, especially caused by the down-regulation of Mfn, is closely related to liver diseases due to an impairment in fatty acid oxidation, the TCA cycle, and glycolysis. Recent studies demonstrated that FUN14 domain containing 2, a mitochondrial protein which is upregulated in human hepatocellular carcinoma, interacted with and inhibited the GTPase domain of MFN1, resulting in the suppression of mitochondrial fusion.^406^ It was also proved that downregulation of Mfn2 occurs in conditions such as liver cancer, non-alcoholic steatohepatitis, and liver fibrosis.^407–410^ Aerobic exercise may benefit the restoration of Mfn2 levels in liver mitochondria and promote fatty acid oxidation, then alleviating the symptoms of non-alcoholic fatty liver disease.^411^

However, in some liver diseases, giant mitochondria are formed due to increased mitochondrial fusion and decreased fission, which is regarded as the structural marker of liver diseases.^412^ For example, in alcoholic hepatitis, alcohol inhibits the expression of Drp1 by impairing hepatic transcription factor EB, thus leading to an increase of giant mitochondria in the liver and appearing liver damage.^413^ Therefore, mitochondrial health is critical to maintaining liver function and preventing disease progression.

### Autoimmune diseases

Autoimmune diseases are owing to the abnormal activity of the immune system, which attacks body tissues and organs, including systemic lupus erythematosus (SLE), rheumatoid arthritis (RA), myasthenia gravis, multiple sclerosis and so on.^414^ The pathogenesis of autoimmune diseases includes genetic factors, cross-antigens, abnormal immune regulation and autoantigens.^415,416^

Mitochondrial dysfunction that triggers inflammation is one possible pathogenesis of autoimmune diseases. mtDNA act as DAMP to activate PRP as well as to induce immune response and inflammation.^417^ Oxidized mtDNA is released into the cytosol via mPTP and VDAC-dependent channels on mitochondria, activating the NLRP3 inflammasome and driving a range of inflammatory diseases.^130^ Less mtDNA copy number and more mtDNA damage are observed in SLE, which is associated with disease durations and prognosis in some SLE patients.^418,419^ In RA patients, the circulating mtDNA copy number was significantly higher than in healthy controls.^420^ One study found that in RA, fat mass and obesity-associated protein-cytidine/uridine monophosphate kinase 2 axis induced mtDNA expression and mediated the cGAS-STING pathway, enhancing synovial inflammation.^421^

Mitophagy inhibits the excessive inflammatory response induced by mitochondrial damage in autoimmune diseases.^422^ In SLE, the overexpression of CD38 impairs the PINK-Parkin pathway and disrupts mitophagy, leading to mitochondrial structural defects in SLE patients.^423^ Stimulating mitophagy has been shown to alleviate lupus nephritis and reduce inflammatory cell infiltration in the kidney.^424^

### Ocular diseases

#### Age-related macular degeneration (AMD)

AMD usually occurs in the elderly and is characterized by lesions of retinal pigment epithelium (RPE) cells.^425,426^ Aging, smoking, cardiovascular disease, obesity, high-fat diet, hypertension, excessive sun exposure, and other factors can induce mtDNA damage and increase ROS production, resulting in mitochondrial dysfunction and ultimately leading to RPE cell damage.^427^

In RPE cells, the key factor of oxidative stress is the antioxidant enzyme SOD, which was an important member of mitochondrial antioxidation. The SOD absence accompanied with the swells of mitochondrial structure and mitochondrial cristae changes, further decreased ATP production and elevated level of oxidative DNA damage can be observed which eventually impact the RPE function.^428^

On another hand, mitophagy could maintain the balance of oxidation and antioxidation through dynamically removing damaged mitochondria, thus avoiding excessive ROS accumulation and oxidative stress. RPE cells in AMD were observed with decreased PINK1 mitophagy pathway, affecting mitophagy and increasing ROS, which induces epithelial-mesenchymal transition.^429^

Many studies focused on mitochondrial function and RPE cell senescence in AMD.^430^ Changes of mitochondrial structure and size exhibit in elderly RPE cells. Mitochondrial dynamics regulates cellular oxidative stress by regulating cellular ROS levels and maintaining mitochondrial function. In aged mice, the Drp1/Mfn2 ratio was increased in RPE-choroid complex, indicating the occurrence of mitochondrial fission.^431^ Yu et al. found that phosphoglycerate mutase (Pgam) 5 is also involved in cell senescence, which is verified to stimulate mitochondrial fission. In *Pgam5*-deleted mice, it counterproductively promoted mitochondrial fusion, leading to intracellular ROS accumulation as well as oxidative stress, thus accelerating RPE cell senescence, in which the IRF/IFN-β pathway was also observed, which may be related to cell senescence.^432^

#### Diabetic retinopathy (DR)

DR is a common microvascular complication of diabetes that predominantly targets the retina. Hyperglycemia induces retinal metabolic abnormalities through various pathways, including the polyol pathway, hexosamine pathway, aging, PKC activation, angiotensin II (ANG-II) pathway et al. These abnormal retinal metabolisms further exacerbate damage, leading to the occurrence of DR.^433,434^ Additionally, hyperglycemia triggers activate oxidative stress and inflammation, along with mitochondrial dysfunction, drawing significant attention from several researchers.

Diabetes promotes raised mitochondrial damage and ROS production to retinal microvessels, leading to corresponding inflammation and oxidative stress which induces DR.^434–436^ During hyperglycemia, retinal mitochondria experience alterations in mitochondrial membrane potential and an increase of membrane permeability, leading to mitochondrial swelling.^437^ In type 2 diabetic rats, more mtDNA injury and less mtDNA copy number were observed in retinal microvessels.^438^ Damaged mitochondria induce oxidative stress. In the diabetic retina, oxidative stress increased and MnSOD attenuated, indicating the imbalance between oxidation and antioxidation.^439^

Impairment of mitochondrial dynamics is one of the mechanisms by which hyperglycemia harms mitochondria homeostasis.^440,441^ In diabetic rats, impaired mitochondrial fusion due to decreased OPA1, and more mitochondria fragmentation in retinal vascular cells were observed.^442^ In addition, OPA1 lowering in the retina of diabetic mice also induced the expression of the pro-apoptotic gene Bax and the release of cytochrome c, which stimulated the apoptosis of retinal vascular cells.^443^ Drp1, which has the opposite function to Opa1, is overexpressed in retinal capillaries of diabetic mice, and such upregulation similarly increases Bax expression and promotes caspase-3 activation.^444^ Many studies suggest that high glucose triggers increased mitochondrial fission in retinal capillaries, which induces cell death through the activation of pro-apoptotic genes and other methods, thereby triggering diabetic retinopathy.

High glucose significantly increased ROS production in RPE cells and decreased PINK1 and Parkin expressions in RPE, thus inhibiting mitophagy.^445^ In diabetic retinopathy, mitophagy is often inhibited, leading to the cumulation of damaged mitochondria, and finally inducing the disease.^445^ Zhang et al. confirmed that the up-regulation of Drp1 induced by high glucose promoted the release of hexokinase-II from mitochondria, which subsequently inhibited the PINK1/Parkin pathway of mitophagy, leading to retinal damage in DR.^446^ Another study exhibited that G-protein-coupled bile acid receptor 5 reversed the growth of intracellular calcium triggered by high glucose, consequently inhibiting Drp1-mediated mitochondrial fission and enhancing the PINK1/Parkin mitophagy pathway.^447^ Zhou et al. observed that notoginsenoside R1 (NGR1), an extract of *Panax notoginseng*, could upregulate mitophagy and reduce mtROS, inhibiting oxidative stress and alleviating diabetic retinopathy by increasing PINK1 and Parkin expression when high glucose.^448^ Therefore, drugs that target inhibiting mitochondrial fission and promoting mitophagy may be a promising direction for DR treatment.

#### Glaucoma

Glaucoma is a neurodegenerative disease characterized by retinal ganglion cells with irregulated structure and function.^449,450^ The main lesions in glaucoma increased intraocular pressure, vascular ischemia, and hypoxia in the optic nerve head. Increased intraocular pressure altered mitochondrial morphology in the trabecular meshwork.^451^ Hypoxia reduces the amount of oxygen, shifts metabolism from OXPHOS to glycolysis, and therefore decreases ATP production in mitochondria. The reduction of ATP as well as the increased ROS will both contribute to mitochondrial dysfunction, which in turn conduce to oxidative stress and inflammation.^452,453^ In neurodegenerative diseases, neurotoxic substances from neuronal damage help to activate Drp1-mediated mitochondrial fission in microglia, down-regulate OXPHOS and ATP production with elevated ROS production, meanwhile motivating inflammasome formation.^454^ Such damage can be transmitted to astrocytes and trigger more inflammatory factors to activate inflammatory responses.^454^ In glaucoma mice, mitophagy may also be downregulated, and the mitophagy marker Rheb was observed to be weakened in the retina,^455^ so the damaged mitochondria are difficult to be cleared by mitophagy.

#### Others

Due to the direct contact with the outside, the anterior eye structure is prone to oxidative stress, inflammation, and other lesions, which may lead to many anterior eye diseases such as keratopathy, conjunctiva disorders, dry eye, etc.^456,457^

The cornea plays a protective and refraction effect. Dry eye is one of the most common corneal diseases, which can result in ocular pain and even visual damage.^458^ Ocular surface inflammation, pro-inflammatory factors, NETs, extracellular DNA and oxidative stress are involved in the development of dry eye diseases.^459–462^ Therefore, mitochondrial dysfunction may contribute to the occurrence and deterioration of dry eye.^463^ Recently, a research identified that in human corneal epithelial cells exposed to hyperosmotic stress, mtDNA passed through the mPTP pore and was released to the cytoplasm, which activates the cGAS-STING pathway and aggravates inflammation.^464^

Corneal alkali burns, a corneal trauma, may result in blindness, while conservative treatments are often ineffective. Zhang et al. reported that the expression of Drp1 climbed and reached a peak on day 3 after alkali burn, with a continuous reduction of Mfn2 expression from day 1 to day 7, resulting in up-regulated mitochondrial fission.^465^ In this process, IL-1β and TNF-α were also up-regulated and promoted the inflammatory response, but such inflammation could be alleviated by Drp1 inhibitor and the mitochondria-targeted antioxidant.^465^

Keratoconus is a corneal disorder with a corneal cone-like protrusion.^466^ In keratoconus patients, the oxidation and antioxidation of corneal epithelium are dysregulated.^467–469^ Yildiz et al. showed that PINK1 expression was down-regulated in corneal epithelium, suggesting that mitophagy may take part in the pathogenesis of keratoconus.^470^

The eyelid is an important structure to protect the eyeball, which can prevent the eyeball from being damaged by external light and foreign matters. The closure of the eyelid also evenly moistens the cornea and prevents corneal dryness. However, the eyelid is directly exposed to the outside, making it more susceptible to inflammation, such as meibomian gland inflammation.^471^ Bu et al. uncovered that chronic inflammation of the meibomian gland was accompanied by the destruction of mitochondrial structure, especially mitochondrial cristae, which suggests that mitochondrial damage might participate in the inflammatory response mechanism of the meibomian gland.^472^

## Therapeutic advances

Since mitochondria affect disease progression in a variety of ways, emerging studies have been focusing on mitochondria-targeted therapies. Many sites of mitochondria serve as potential targets for mitochondrial therapy, including respiratory chain, oxidation and antioxidation, mitochondrial dynamics, and so on. Other treatments, including lifestyle improvements, mitochondrial transplantation, photobiomodulation, and photodynamic therapy, can also benefit health by modulating mitochondrial function. Additionally, with the striking development of material science especially biomaterials for drug delivery, effective and targeted therapy with extremely low side effects attracts great attention. In this review, we summarize the mainstream mitochondria-targeted therapy, along with their mechanisms and advances.

### Exercise and diet

Exercise affects mitochondrial number and function by regulating mitochondrial biogenesis, mitochondrial dynamics, and mitophagy. Exercise increases ATP consumption and therefore activates AMPK signaling pathway.^473,474^ AMPK activate peroxisome proliferator-activated receptor coactivator-1α (PGC-1α), the key factor in mitochondrial biogenesis, thus enhancing mitochondrial respiratory and metabolic functions.^475,476^ In a study that included eight participants, the researchers let the experimental group perform twice-daily exercise, which was a 2-h muscle glycogen-depleting exercise followed by a 2-h rest and then the high-intensity interval exercise, and these twice-daily exercisers had a significant increase in the expression of PGC-1α, compared to the once-daily and control groups.^477^ However, in another similarly-designed study, training twice a day enhanced mitochondrial efficiency, but did not seem to significantly increase the protein abundance of PGC-1α compared to once a day.^478^ AMPK also promotes the mitochondrial fission protein Drp1 sometimes, thereby affecting mitochondrial dynamics.^242,479^ The aging process is proven to be accompanied by inhibited AMPK pathway, as well as decreased physical fitness and mitochondrial fragmentation.^480^ In addition, exercise enhances mitophagy and restores mitochondrial function that is impaired in aging or disease by elevating Parkin and PINK1 levels.^481,482^

The quantity, intake timing, nutrient composition, and microbial regulation of food consumption all affect health outcomes. According to a clinical study, three dietary intervention methods, including caloric limitation, intermittent fasting, and a ketogenic diet, positively intervene in mitochondrial respiratory function and increase cellular oxygen consumption rates.^483^ Caloric limitation, intermittent fasting and specific amino acid restriction are all found to be able to extend lifespan.^484^ Aging and aging-related diseases are characterized by a decline in metabolism, and dietary changes can intervene in this decline. In a randomized controlled trial, 11 obese participants received a dietary intervention that reduced their intake by 1,000 kcal, after 16 weeks their peripheral insulin sensitivity enhanced.^485^ Long-term dietary restrictions are beneficial in controlling inflammation. In a randomized controlled trial, participants were subjected to 12% calorie restriction, and their RNA sequences showed that up-regulated genes compared to controls were mainly enrichened in pathways such as autophagy, DNA repair, circadian rhythms, and mitochondrial biogenesis, whereas the inflammatory pathways were down-regulated.^486^

### Mitochondria-targeted drugs

#### NAD supplements

NADH is the raw material for superoxide production in mitochondria. NAD supplements are beneficial in improving energy metabolism, maintaining mitochondrial function, aiding DNA repair, and anti-aging. Oral nicotinamide riboside (NR) supplementation has been shown to be safe in some studies.^487^ In randomized trials, continuous oral administration of NR has been concluded to increase the NAD metabolome.^487,488^ Furthermore, NR has been shown to down-regulate inflammatory factors and suppress inflammatory responses in heart disease.^489^

However, oral administration of NR would lead to a lower bioavailability due to the difficulty of crossing barriers and the degradability when encountered in vivo circumvents. Nanosized drug delivery systems offer a potential solution to this problem. Nanomedicine significantly enhances drug bioavailability by reducing particle size to the nanoscale, which facilitates them to pass through biological barriers.^490–492^ Additionally, they are feasible to be engineered with specific modifications to accurately target lesion sites and realize prolonged retention at the exact locations.^493,494^ Furthermore, the introduced nanosized carriers could give protection to the loaded therapeutics that significantly extended the function duration with reduced degradation speed in vivo.^495^ For example, in the treatment of ocular diseases, traditional eye drops must navigate multiple anatomical barriers, including the tear film, cornea, Bruch membrane, and blood-retinal barrier, resulting in poor permeability and limited therapeutic efficacy.^496^ Ocular activities, such as tear flushing and blinking, further shorten the residence time on the eyes.^497^ Intriguing points lie in that the nano-based eye drops could improve drug delivery to specific ocular tissues by increasing residence time, enhancing barrier penetration, and effectively targeting specific sites.^498^ Therefore, Nie et al. used natural polyphenol resveratrol to assemble with NR into microspheres, with remarkably elevated NR bioavailability in various organs, such as heart, brain and kidney, and alleviated ischemia-reperfusion injury in mouse heart is observed.^499^ Similarly, Duan et al. chose Fe_3_O_4_ nanoparticles to carry nicotinamide mononucleotide, the precursor of NAD, which could promote the delivery of nicotinamide mononucleotide to renal cells, down-regulate the level of IL-6 and finally alleviate renal ischemia-reperfusion injury.^500^

#### Antioxidants

Antioxidants prevent the damage of ROS by reducing free radicals, oxidation reactions and so on, which play a vital role in protecting cells and improving mitochondrial function. In recent years, mitochondria-targeted antioxidants have attracted much attention. Targeting antioxidants to mitochondria can improve their efficiency and directly suppress the oxidation damage at its origin.

MitoQ is one of the most popular mitochondria-targeted antioxidants. It is a derivative of coenzyme Q10, helping its selective accumulation within mitochondria to maintain and restore mitochondrial function. MitoQ supplementation could reduce nuclear DNA and mtDNA damage.^501^ MitoQ enhances the activity of antioxidant enzymes in mitochondria and alleviates oxidative stress.^502,503^ It also contributes to inhibiting inflammatory responses by reducing mtROS level, suppressing the NLRP3 inflammasome, and promoting M2 microglia polarization.^504^ Moreover, mitoQ aids in slowing aging by alleviating oxidative stress and inflammation, improving metabolism, and protecting mitochondrial function. For example, it can attenuate melanocyte senescence and subsequent skin aging.^505^ By alleviating smoking-induced oxidative stress and alveolar epithelial cell senescence, MitoQ could alleviate lung injury.^506^ It also exerts beneficial effects in improving cardiovascular function,^507^ alleviating ischemia-reperfusion injury,^508,509^ and inhibiting tumor recurrence and metastasis.^510,511^

Increasingly research focused on the incorporation of nanotechnology with antioxidants to treat mitochondria-related diseases. Compared with traditional therapy modalities, nanoparticles improve the targeting efficiency of mitochondria, enhance the stability and bioavailability of therapeutics, and reduce the side effects on other non-target sites. A few researchers have made an attempt to encapsulate MitoQ in nanoparticles to form nMitoQ, which realizes the reduction in oxidative stress and acts as an anti-inflammatory agent. nMitoQ has been identified to improve placental mitochondrial function in rats exposed to hypoxia during pregnancy, with significantly elevated complex IV activity in the placentas of male offspring.^512^ Meanwhile, nMitoQ is proven to reduce the superoxide levels in pregnant hypoxic placentas and to promote placental angiogenesis in female placentae by upregulating the expression of VEGF.^513^ Cardiovascular dysfunction is one important fetal defect brought about by prenatal hypoxia, which may lead to fetal growth restriction and even death. In prenatally hypoxic rats, being treated with nMitoQ could suppress the rise in ROS and attenuate the heart diastolic dysfunction, especially in female progeny^514^ Furthermore, improved cardiac tolerance after adulthood could be seen in the offspring of these prenatal hypoxic pregnant rats administrated with nMitoQ.^515^

In addition, as many new materials have been identified to act as antioxidants, their unique characteristic endowed them as promising candidates in medical fields. Selenium is an essential element that makes up GPx and is important for GPx to exert appropriate antioxidant functions. In a notable study, selenium nanoparticles exhibit higher bioavailability and antioxidant capacity compared to traditional selenium. Moreover, significantly increased GPx activity in Parkinson’s disease rat models was observed with the administration of selenium nanoparticles, along with improved antioxidant capacity and relieved oxidative stress.^516^ Other metal nanoparticles, such as silver and platinum nanoparticles, the ability to scavenge superoxide and then display antioxidant capacities.^517^ While silver ions are well known for their antibacterial ability, the antioxidant capacity of silver nanoparticles has also received much attention. Silver nanoparticles can be prepared via physical, chemical and biological methods, among which biosynthetic one is the most popular.^518^ Those biosynthesized silver nanoparticles are rich in bioactive compounds such as polyphenols and flavonoids, which gives better antioxidant capacity.^518^ As previously mentioned, nanomaterials are more effective in passing through the ocular barriers and prolonging drug retention in the eyes. Mitochondria-targeting nanosized nanomaterials attract great attention in the ocular drug delivery systems. For example, the aging-related loss of melanin in RPE, an antioxidant, that contributes to the accumulation in ROS, is regarded as one of the inducements of AMD.^519,520^ Kwon et al. demonstrated that the intravitreal injection of synthetic melanin-like nanoparticles can clear free radicals, with reduced ROS production in RPE cells, finally alleviate the vascular leakage and show the anti-angiogenic activity in an AMD-like model.^520^ Glaucoma is associated with obstructed outflow of aqueous humor and elevated intraocular pressure. Curcumin has antioxidant and anti-inflammatory effects. Cheng et al. developed a nanoparticle drug delivery system loaded with curcumin and latanoprost, which effectively inhibit the expression of inflammatory factors and reduce ROS production in trabecular meshwork cells, proving its certain potential for the treatment of glaucoma.^521^

#### Mitochondrial dynamics regulators

Mitochondrial dynamics not only manages the number and size of mitochondria but also affects mitochondrial ROS production and mitochondrial metabolism.

Silibinin is a flavonolignans component extracted from silymarin, which has anti-inflammatory, anti-infective, and anti-tumor effects.^522^ Silibinin exhibits efficacy in the treatment of breast cancer, lung cancer, and skin cancer.^522–524^ In vitro results demonstrated that mitochondria tend to form smaller and more fragmented ones in breast cancer cells treated with silibinin, indicating altered mitochondrial dynamics. Subsequently, the expression of Drp1 and Fis1 was up-regulated, while down-regulated expression of Opa1 and Mfn1 was observed, which indicated the effect of silibinin to enhance mitochondrial fission and to weaken mitochondrial fusion.^525^ As a result of promoted mitochondrial fission, excessive mitochondrial fission will eventually lead to mitochondrial damage, followed by apoptosis. Therefore, the treatment of tumors using silibinin may benefit from the characteristic to induce apoptosis of tumor cells.

Recently, some nanomaterials were discovered to regulate mitochondrial dynamics, hence their unique sizes enable them to easily enter cells and are more likely to interact with intracellular molecules, which may affect mitochondrial dynamics. Nanoparticles affect mitochondrial dynamics by intervening in various mitochondrial dynamics-related proteins. For example, silver nanoparticles upregulate the level of Drp1, disrupting mitochondrial structure and function.^526^ When exposed to silver nanoparticles, there was also a rising expression of the Fis1 and an inhibited expression of OPA1, which led to excessive fission and apoptosis.^527^

#### Mitophagy regulator

Activation of mitophagy promotes the removal of damaged mitochondria and avoids excessive ROS production, as well as the corresponding oxidative stress and inflammation. Urolithin A is a mitophagy activator, which is verified to regulate mitophagy through the PINK1/Parkin pathway. Improved mitochondrial oxidative metabolism and reduced inflammatory factors can be obtained through oral administration.^528,529^ Metformin, a traditional diabetes drug, upregulated the expression of mitophagy genes, such as *PINK*, *Parkin*, and *Mfn2*, while at the same time effectively reducing damaged mitochondria after administration, indicating a great potential of metformin as a mitophagy activator.^530^

Some nanomedicine, especially metal oxide nanoparticles have been reported to modulate mitophagy via affecting critical mitophagy-related proteins. In cell experiments, zinc oxide nanoparticles significantly elevated the PINK1, Parkin, and LC3 expression levels, promoted autophagosome formation, resulting in cell cycle arrest, and inhibited tumor cell proliferation in diffuse large B-cell lymphoma, potentially explaining their anti-tumor effect.^531^ In osteosarcoma cells, zinc oxide nanoparticles can also be observed to increase the expression of HIF-1α, mediate mitophagy through the HIF-1α-BNIP3-LC3B pathway, and inhibit tumor growth,^532^ along with tumor cell metastasis.^533^ In neuroblastoma cells, zinc oxide nanoparticles not only up-regulated PINK1, Parkin, and Drp1, but also decreased Mfn2, and Opa1, indicating that zinc oxide nanoparticles could also change mitochondrial dynamics.^534^ However, with the increasing attention paid to ZnO nanoparticles, it should be noted that ZnO nanoparticles may also cause cytotoxicity in normal cells. For example, when pregnant mice were exposed to ZnO nanoparticles, ZnO nanoparticles could pass through the blood-brain barrier, and reach the fetal brain, causing microglial inflammation and eventually leading to nervous system damage in fetal mice.^535^

#### MTP131

Elamipretide (MTP131) is a tetrapeptide targeted to the inner mitochondrial membrane, reducing ROS production, stabilizing enzyme activity, and improving mitochondrial energy production. It was tested that MTP131 can improve exercise ability in patients with mitochondrial myopathy.^536^ The addition of MTP-131 to the calycosin/tanshinone nanosystem significantly reduced infarct size in rats with acute myocardial infarction, showing promising treatment outcomes.^537^

### Mitochondria transplantation

Mitochondrial transfer between cells is an important way to maintain intercellular homeostasis, which can also help to rescue damaged cells or give them new properties. As early as 1982, Clark and Shay found that after mitochondria isolated and purified from antibiotic-resistant cells were transferred to antibiotic-sensitive cells, these antibiotic-sensitive cells acquired stable antibiotic resistance.^538^ The modes of intercellular mitochondrial transfer that have been identified are tunneling nanotubes, exosomes, direct release and capture.^539,540^ Mitochondrial transplantation is an emerging treatment for diseases linked with mitochondrial dysfunction in which healthy mitochondria are isolated and transplanted back into tissues or organs to enhance the function of damaged cells.^541^

In animal studies, there have been many studies that support mitochondrial transplantation for improved function of damaged cells. Hayashida et al. intravenously injected frozen-thawed or fresh mitochondria into mice resuscitated from cardiac arrest, it was found that injection of fresh mitochondria elevated ATP content and increased survival from 55% to 91% at 72 h after resuscitation from cardiac arrest.^542^ The safety of mitochondrial transplantation was evaluated in a clinical trial that included 30 patients with myocardial infarction, in which participants in the treatment group accepted autologous platelet-derived mitochondria injections into the coronary arteries, and after 40 days, the treatment group had improvements in left ventricular ejection fraction and exercise capacity, but there were no significant differences in the occurrence of adverse events such as arrhythmias and fever between the treatment and control group.^543^

Nonetheless, clinical trials of mitochondrial transplantation remain inadequate. The first thing to consider is the technical issue of how to transplant healthy mitochondria more efficiently into the target tissue to ensure maximum treatment impact. Second, the security issue needs further validation. More clinical studies are still needed to evaluate the safety and feasibility of mitochondrial transplantation for clinical applications. Especially when the source of transplanted mitochondria is exogenous, whether mitochondria as an exogenous substance causes an immune response is something to think about and verify.

### Mitochondria photobiomodulation

Photobiomodulation (PBM) is a therapy that uses low-intensity laser light to stimulate tissue or cells. The laser light usually has the wavelength within 600 to 1000 nm, and is commonly red or near-infrared laser light. Mitochondria are key target of PBM since the laser is absorbed by mitochondria. In pathological states, when irradiated by laser light, cytochrome C oxidase in the mitochondria absorbs light energy, which strengthens the activity of the electron transport chain, and the mitochondrial membrane potential increases, promoting ATP synthesis and energy supply.^544^ Some studies have shown that, low fluence PBM is accompanied by an appropriate amount of ROS production, and these low levels of ROS can promote the activation of the antioxidant system activity and regulate the ROS balance.^545^ However, high fluence PBM will lead to excessive ROS production.

PBM is thought to have anti-inflammatory effects by reducing inflammatory markers in cells. Several clinical trials have showed that PBM help patients reduce inflammation and relieve pain. In a clinical trial of 13 participants receiving dental implants, half of whom received twice PBM treatments after implanting, the laser used a combination of infrared light at 808 nm and red light at 630 nm, and the results showed that the intervention group that received PBM healed better, with inflammation occurring in only 35% of cases, compared to 70% in the control group.^546^ Another clinical trial that included 55 patients undergoing oral cavity and oropharyngeal squamous cell carcinoma showed that receiving PBM during radiotherapy delayed the onset of oral and oropharyngeal mucositis and provided pain relief.^547^ Furthermore, an animal experiment revealed that treating aged mice with PBM at a wavelength of 650 nm increased antioxidant enzyme activity while decreasing levels of IL-6 and TNF-α, as well as improving mitochondrial function in aged mice’s ovarian cells, implying that PBM has the therapeutic potential to alleviate ovarian aging.^548^

### Mitochondria photodynamic therapy

Photodynamic therapy (PDT) is a treatment centered on photosensitizers, light, and oxygen. Mitochondrial PDT involves targeting photosensitizers to the mitochondria, and when light excites the photosensitizers, the photosensitizer undergoes an energy transfer to produce a lot amount of ROS. These ROS are destructive, damaging the cellular structure and leading to cell death, therefore PDT commonly used in the treatment of tumors. Zhou et al. found that PDT treatment of colorectal cancer cells with a photosensitizer that specifically targets mitochondria induced elevated levels of mitochondrial ROS in the cells and induced colorectal cancer cells pyroptosis via the caspase 3/gasdermin E pathway.^549^ Besides, since photosensitizers often have hydrophobic properties that limit transport, nanomaterials combined with photosensitizers can better help the photosensitizer to localize in mitochondria. Chen et al. designed a mitochondria-targeted PDT nanoplatform and showed that this nanoplatform could be well localized in the mitochondria, generating more ROS and causing more severe cytotoxicity.^550^ In addition to tumor therapy, some clinical studies have shown that PDT is also used in the treatment of skin diseases and periodontitis, PDT may have broader applications in the future.^551,552^

### Clinical progress and challenges

At present, there are more and more clinical trials regarding mitochondria-targeted therapies, covering different disease directions, especially diseases related to mitochondrial dysfunction, such as cancers, motor muscle diseases, neurodegenerative diseases, cardiovascular diseases, metabolic diseases, and rare mitochondrial diseases.^483,489,528,553^ Bielcikova et al. investigated the clinical effects of mitochondrial targeting tamoxifen in a phase I/Ib single-center trial recruiting patients with metastatic tumors.^554^ The trial found that this mitochondrial-targeted therapy achieved a clinical benefit rate of 37%, with the most significant impact observed in patients with renal cell carcinoma, and the main adverse reaction was haematological toxicity in the phase I trial.^554^ Other complications observed in this trial include fever, anemia and hromboembolic complications.^554^ As one of the most concerned mitochondrial-targeted antioxidants, many clinical studies focus on MitoQ. A pilot clinical study in 2023 demonstrated the effect of MitoQ on microcirculation in patients with chronic kidney disease.^555^ Participants were treated with either MitoQ (20 mg/day MitoQ) or placebo. It was subsequently found that MitoQ treatment improved microcirculatory function without affecting blood pressure.^555^ In a phase II study, after 28 days of continuous oral administration of Mito-Q in patients with chronic HCV infection, their liver function was found to be improved.^556^ In recent years, more and more attention has been paid to the individual differences and safety of Mito-Q treatment. The effect of Mito-Q varied among populations. In a randomized, controlled, double-blind study by Carlini et al., alterations in endothelial function and cardiopulmonary fitness were found to be different in middle-aged and elderly people treated with 80 mg of Mito-Q.^557^ Besides, Mito-Q improved endothelial function in patients with brachial artery flow-mediated dilatation<6%, but not vice versa.^557^ Given the clinical safety profile of Mito-Q, Linder et al. explored whether high doses of Mito-Q could cause nephrotoxicity.^558^ Therefore, they designed a randomized crossover experiment involving 32 healthy adults taking 100–160 mg Mito-Q or placebo, and after 4–6 h urine results showed no significant differences between these two groups.^558^ However, this trial showed only the short-term safety of acute high-dose Mito-Q, another clinical trial evaluated the long-term safety of Mito-Q. Rossman designed a randomized controlled trial involving 20 elderly people taking 20 mg/d Mito-Q or placebo for 6 weeks, and the results showed that the Mito-Q group was well tolerated without serious adverse events.^507^ Although these studies have shown the safety of Mito-Q in long-term use, the safety assessment of a longer scale has not been fully studied. Mitochondria are highly interconnected organelles, which involve many pathways. We still need more and longer-term clinical studies to pay attention to whether mitochondrial targeted therapy will bring adverse reactions.

## Conclusion and prospect

As mentioned above, mitochondria have a close interaction with oxidative stress, inflammation, and aging. Mechanistically, mitochondria participate in the energy metabolism, signal transduction, and intercellular communication of cells. By changing the morphology, size, electron transport chain, cell membrane receptors, enzymes, and other mitochondrial properties, mitochondria can adapt to different environments, cope with different challenges, and play an appropriate role. In this review, we sum up the interactions of mitochondria with oxidative stress, inflammation, and aging, as well as the mitochondrial changes in diseases and mitochondria-targeted drugs.

However, the clinical application of mitochondria still faces some challenges. First, although mitochondrial function is related to oxidative stress, inflammation and aging, exactly how mitochondria specifically coordinate with other cellular activities to drive each type of disease still warrants further investigation. Secondly, in addition to the role of mitochondria mentioned above, mitochondria may also have an impact on other physiological and biochemical activities, and whether these links are related to oxidative stress, inflammation, and aging is still under debate. Third, although emerging studies have been carried out on the therapy targeted mitochondria, there is still a long journey from the research to clinical trials and the eventual clinical application. Additional research is required to determine whether or not it can play a similar role in clinical patients. Clinical trials are essential before new drugs enter the market, and some drugs may not play a better role in humans or may be restricted as non-negligible side effects.

In a world burdened by diseases associated with oxidative stress, inflammation, and aging, it is crucial to identify a focal point for intervening in related diseases. Mitochondria serve as a central hub, which plays a vital role in linking the above-mentioned key factors. Moreover, drugs targeting mitochondria also contribute to the diagnosis and treatment of diseases. At present, multidisciplinary integration has brought important breakthroughs for disease prevention and treatment, but more forces are still needed to solve the problem. As mentioned in this review, the application of nanomaterials opens a breakthrough for the clinical application of drugs targeting mitochondria. In the field of future mitochondrial-targeted drug development, several challenges remain. Mitochondria have a bilayer membrane structure, which makes it difficult for some traditional drugs to penetrate. In addition, due to the central role of mitochondria, their regulation is inextricably linked. Attention to other side effects induced by mitochondrial-targeted therapy is warranted. Moreover, mtDNA and nuclear DNA in the mitochondrion cause it to receive the regulation of two sets of genetic information, and may also get the mutation of two kinds of genome, making the treatment more complicated.

Nanomedicine targeting mitochondria is the key to solve this problem. Through the precise delivery of drugs to mitochondria, it can effectively interfere with energy metabolism, oxidative stress, and cell homeostasis. Although some nanomedicines have shown potential mitochondrial targeting, nanomedicines still face several challenges from experimental to clinical practice. The first issue is biosafety. The current mainstream mechanism of targeting mitochondria by nanomedicine focuses on the adsorption of positive charges to the mitochondrial membrane.^559^ However, excessive positive charge can depolarize the mitochondrial membrane and trigger a series of oxidative stress or inflammatory responses.^559^ In addition, the targeting efficiency of nanomedicine still needs to be improved. Besides, current mitochondria-targeting nanomedicines mainly focus on disease treatment, but the exploration of mitochondria-targeting probes that monitor disease progression is equally important, such research is still insufficient.

In the future, more attention should be paid to the long-term safety and efficacy of targeted drugs in future clinical studies. In addition, with an in-depth understanding of the molecular mechanism of mitochondria, personalized targeted therapy has become the focus of future medical care. Designing appropriate drugs according to the genetic background and disease characteristics of patients can achieve precision treatment and further improve the treatment effect. It is foreseeable that mitochondria-targeted drugs will play a promising role in the precision treatment of many diseases.

Mitochondria are energy-producing organelle, along with the generation of reactive oxygen species in the process. At the same time, the mitochondrion has its mtDNA, which endows it with special abilities, but also makes it more susceptible to damage. In this review, we describe the central roles of mitochondria in oxidative stress, inflammation, and aging, especially mechanisms in diseases, and the current mainstream drugs targeting mitochondria. However, the specific mechanisms by which mitochondria act among the three need to be further explored. In the future, more attention should be paid to the development of drugs targeting mitochondria. Based on the central position of mitochondria, mitochondrial therapies have great potential. However, how to apply it to specific diseases needs more clinical research and data to monitor the actual therapeutic effect. In summary, mitochondria can regulate oxidative stress, inflammation, and aging, and drugs targeting mitochondria can be involved in related diseases. We believe that although clinical research on mitochondria still needs a lot of effort, it is extremely promising in the future.
